# An auxin signaling network translates low-sugar-state input into compensated cell enlargement in the *fugu5* cotyledon

**DOI:** 10.1371/journal.pgen.1009674

**Published:** 2021-08-05

**Authors:** Hiromitsu Tabeta, Shunsuke Watanabe, Keita Fukuda, Shizuka Gunji, Mariko Asaoka, Masami Yokota Hirai, Mitsunori Seo, Hirokazu Tsukaya, Ali Ferjani

**Affiliations:** 1 Department of Biology, Tokyo Gakugei University, Koganei-shi, Tokyo, Japan; 2 RIKEN Center for Sustainable Resource Science, Yokohama, Japan; 3 Department of Life Sciences, Graduate School of Arts and Sciences, The University of Tokyo, Komaba, Meguro-ku, Tokyo, Japan; 4 Laboratoire de Reproduction et Développement des Plantes, Université de Lyon, UCB Lyon 1, ENS de Lyon, INRA, CNRS, Lyon, France; 5 Department of Biological Sciences, Graduate School of Science, The University of Tokyo, Tokyo, Japan; Swedish University of Agricultural Sciences, SWEDEN

## Abstract

In plants, the effective mobilization of seed nutrient reserves is crucial during germination and for seedling establishment. The *Arabidopsis* H^+^-PPase-loss-of-function *fugu5* mutants exhibit a reduced number of cells in the cotyledons. This leads to enhanced post-mitotic cell expansion, also known as compensated cell enlargement (CCE). While decreased cell numbers have been ascribed to reduced gluconeogenesis from triacylglycerol, the molecular mechanisms underlying CCE remain ill-known. Given the role of indole 3-butyric acid (IBA) in cotyledon development, and because CCE in *fugu5* is specifically and completely cancelled by *ech2*, which shows defective IBA-to-indoleacetic acid (IAA) conversion, IBA has emerged as a potential regulator of CCE. Here, to further illuminate the regulatory role of IBA in CCE, we used a series of high-order mutants that harbored a specific defect in IBA-to-IAA conversion, IBA efflux, IAA signaling, or vacuolar type H^+^-ATPase (V-ATPase) activity and analyzed the genetic interaction with *fugu5–1*. We found that while CCE in *fugu5* was promoted by IBA, defects in IBA-to-IAA conversion, IAA response, or the V-ATPase activity alone cancelled CCE. Consistently, endogenous IAA in *fugu5* reached a level 2.2-fold higher than the WT in 1-week-old seedlings. Finally, the above findings were validated in *icl–2*, *mls–2*, *pck1–2* and *ibr10* mutants, in which CCE was triggered by low sugar contents. This provides a scenario in which following seed germination, the low-sugar-state triggers IAA synthesis, leading to CCE through the activation of the V-ATPase. These findings illustrate how fine-tuning cell and organ size regulation depend on interplays between metabolism and IAA levels in plants.

## Author summary

How leaf size is determined is a longstanding question in biology. In the simplest scenario, leaf size would be a function of cell number and size. Yet, accumulating evidence on the model plant *Arabidopsis thaliana* suggested the presence of compensatory mechanisms, so that when the leaf contains fewer cells, the size of each cell is unusually increased (the so-called compensated cell enlargement (CCE)). While decreased cell numbers in the compensation exhibiting *fugu5* mutants have been ascribed to reduced sugar biosynthesis from seed oil reserves, molecular mechanisms underlying CCE remain ill-known. Recently, IBA (a precursor of the phytohormone auxin) has emerged as a potential regulator of CCE. Here, to further illuminate the role of IBA in CCE, we used a series of high-order mutants and analyzed their genetic interaction with *fugu5*. We found that while CCE in *fugu5* was promoted by IBA, defects in IBA-to-auxin conversion, auxin response, or the vacuolar V-ATPase activity alone cancelled CCE. This provides a scenario in which following seed germination, the low-sugar-state triggers auxin synthesis, leading to CCE through the activation of the V-ATPase, illustrating how fine-tuning cell and organ size regulation depend on interplays between metabolism and auxin levels in plants.

## Introduction

Leaves are the primary plant photosynthetic organs and are the site of metabolic reactions critical for survival. In nature, plants have developed survival strategies to adapt to fluctuating environments, including altered leaf size, shape, and thickness. Because floral organs can be considered as modified leaves, understanding leaf development is fundamental for understanding the diverse morphologies found in the plant kingdom [1].

Organogenesis in leaves proceeds through two stages: cell proliferation and cell expansion. Coordination between proliferation and expansion is vital for leaves to grow to a fixed size [2,3]. Many studies have investigated the coordination of cell size and cell numbers in organs of multicellular organisms [4–8]. Among them, studies on *Arabidopsis thaliana* have suggested the presence of compensatory mechanisms in leaves, so that when the leaf contains fewer cells, the size of these cells is unusually increased [3,9–15]. Compensation also suggests that a leaf can perceive its own size, and decreased cell number “input” is translated into excessive cell enlargement “output,” suggesting an important role for cell to cell communication [3,16]. This hints at the existence of a leaf-size regulatory network, and that understanding the molecular mechanism underlying this compensation is key to unveiling the relationship between cell number and cell size in an organ-wide context [17].

Compensation occurs in several mutants and transgenic plants, in which leaf cell numbers are significantly reduced [3,17–19]. Compensation consists of two phases: the induction phase that consists of a reduction in the number of cells due to decreased proliferative cell activity, and the response phase during which post-mitotic cell expansion of individual leaf cells is abnormally enhanced [3,16]. Kinematic analyses have revealed that compensated cell enlargement (CCE) occurs through three different modes: an enhanced cell expansion rate (Class I), an extended cell expansion period (Class II, including *fugu5*), and increased cell size during the proliferative cell stage (Class III) [3,12,17,20–22]. Therefore, to clarify the molecular mechanisms underlying compensation, the induction and response phases must be understood first.

In the Class II compensation exhibiting mutant *fugu5*, mature cotyledons contain ~60% fewer cells, but these cells are ~1.8-fold larger compared to wild type (WT) [3,22–25]. This involves large-scale metabolic modifications. Indeed, in *fugu5*, the loss of the vacuolar H^+^-PPase activity leads to excess cytosolic pyrophosphate (PPi) accumulation, which partially reduces the triacylglycerol (TAG)-to-sucrose (Suc) conversion and cotyledon cell number [23]. During germination, Suc is synthesized from the TAG of the oil bodies *via* β-oxidation, the glyoxylate cycle, the TCA cycle, and gluconeogenesis [26]. Development of *Arabidopsis* seedlings relies on TAG-Suc conversion as the sole energy source before they acquire photosynthetic capacity [26]. Therefore, the *fugu5* mutant exhibits cell proliferation defects in the cotyledons. Consistently, excess PPi interferes with the metabolic reactions that produce Suc, specifically through inhibition of UDP-glucose pyrophosphorylase (UGPase) activity [27].

Although the induction phase in *fugu5* is now better understood, our knowledge of CCE remains limited. We recently reported that the loss of activity of the peroxisomal enzyme enoyl-CoA hydratase2 (ECH2) completely suppressed CCE, not only in the *fugu5* background but also in all Class II mutants, namely, *isocitrate lyase-2* (*icl–2*; [28]), *malate synthase-2* (*mls–2*; [29]), *phosphoenolpyruvate carboxykinase1–2* (*pck1–2*; [30]), hinting at the pivotal role of ECH2 activity in Class II CCE [22,25]. However, ECH2 is involved in many metabolic reactions, leaving the key question of how ECH2 affects CCE, unanswered [31].

ECH2 is partially involved in fatty acid β-oxidation and the conversion of indol-3-butyric acid (IBA) into indol-3-acetic acid (IAA), which is the major endogenous auxin in plant peroxisomes [26,32,33]. IBA is a minor auxin precursor metabolite, while the majority of IAA is synthesized from tryptophan *via* indole-3-pyruvic acid [33–35]. IAA homeostasis is achieved through specific metabolic pathways that can release/store IAA from/into its inactive forms such as amino acids and sugar conjugates, and methyl-IAA [35,36], or synthesize IAA *de novo* from several other metabolic pathways [37]. Although IBA was first detected as a growth-promoting substance in 1954 [38], our understanding of IBA biosynthesis remains fragmentary. Recently, however, INDOLE-3-BUTYRIC ACID RESPONSE (IBR) 1 (IBR1; [39]), IBR3 [40], and IBR10 [39], which are peroxisomal enzymes involved in the reactions synthesizing IAA from IBA, have been elucidated and it has been revealed that IBA is also involved in the regulation of plant development [34,41]. The *ibr1–2 ibr3–1 ibr10–1* triple mutant (*ibr1*,*3*,*10*, hereafter) displayed abnormal root hairs and lateral roots, suggesting a role for IBA in root development [33,34]. On the other hand, it has been reported that the *ibr1*,*3*,*10* mutant displays small cotyledons [34]. More interestingly, mutants with defects in the IBA efflux carriers PENETRATION3 (PEN3; [42]) and PLEIOTROPIC DRUG RESISTANCE 9 (PDR9; [42–44]) have larger cotyledons, when compared to the WT, possibly due to high intracellular IBA, and thus higher endogenous IAA levels in these organs [42]. From these findings, IBA-derived IAA plays an important role not only in roots but also in plant shoots and particularly in cotyledons during their early developmental stage.

In recent years, mutants involved in the production or storage of IBA have been found to display larger or smaller cotyledons, respectively. Based on the involvement of IBA in cotyledon development, we have proposed that IBA might be a key metabolite driving CCE [22,25]. Also, treatment with IAA (1 μM) causes a significant increase in the volume of red beet taproot vacuoles [45,46]. Thus, to elucidate the mechanism of class II CCE, we performed molecular genetic analyses in a *fugu5* mutant background combined with mutants with defects in IBA-to-IAA conversion, IBA efflux, IAA signaling, or V-ATPase activity. Collectively, our findings suggest that CCE in *fugu5* mainly depends on endogenous IAA levels. We identify a strong link between carbohydrate metabolism and plant hormonal signaling, where AUXIN RESPONSE FACTOR (ARF) 7 and ARF19 play pivotal roles in transducing auxin signals and triggering CCE, probably through the activation of the vacuolar V-ATPase.

## Results

### *ibr1*,*3*,*10* mutations completely suppress CCE in the *fugu5* background

In a previous study, we found that CCE was completely suppressed in *ech2–1 fugu5–1* and *ech2–2 fugu5–1* mature cotyledons, suggesting that IBA, a substrate of ECH2, is likely involved in *fugu5* CCE [22]. However, Li *et al*. [31] found that the accumulation of the precursor compound of the metabolic reaction catalyzed by ECH2 caused *ech2–1* developmental defects. Simultaneously, this metabolic disorder in *ech2–1* also indirectly affected the conversion of IBA to produce IAA. These findings indicate that the loss of ECH2 affects many metabolic reactions, including IBA-to-IAA conversion. Moreover, the triple mutant *ibr1*,*3*,*10* exhibits a typical low-auxin phenotype reminiscent of *ech2–1* [33]. Based on these studies, we conducted genetic analyses using *ibr1*,*3*,*10 fugu5–1* to corroborate the importance of IBA-to-IAA conversion in CCE.

**Fig 1:**
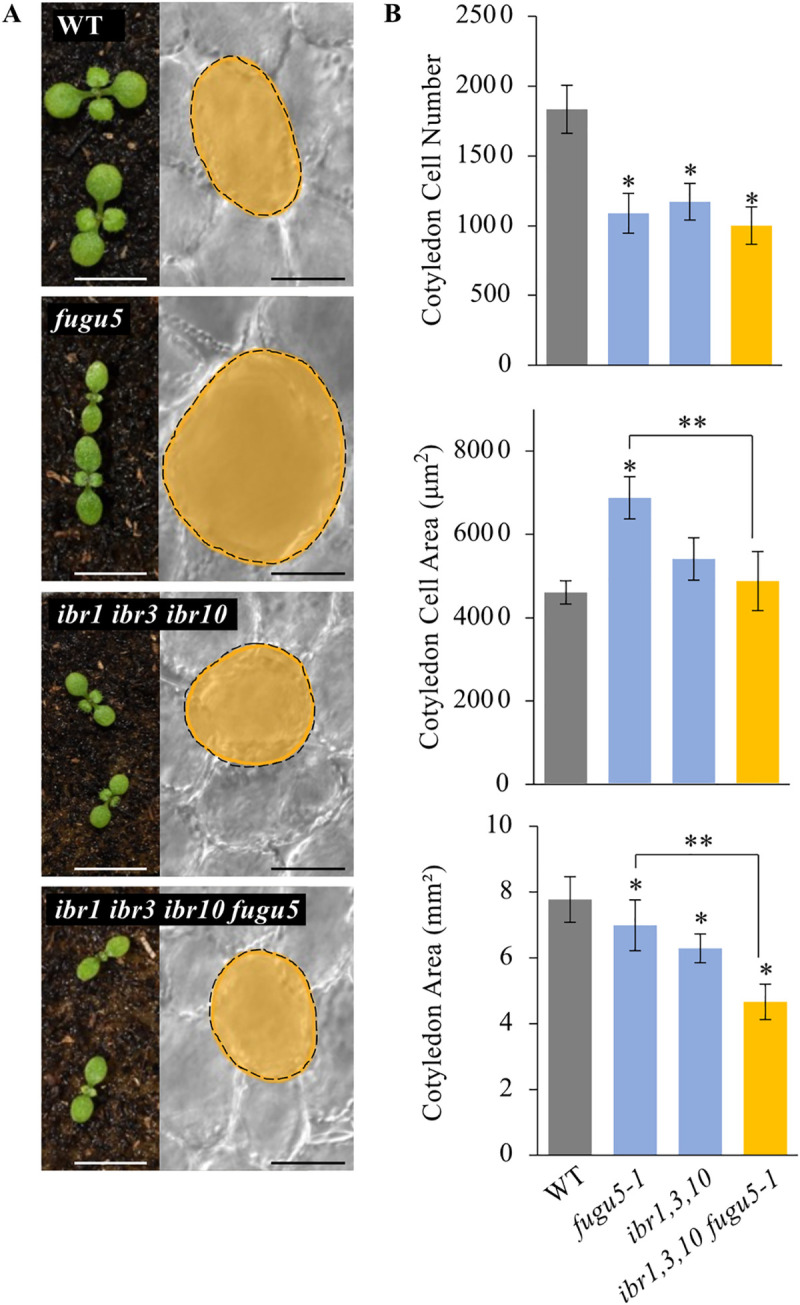
*ibr* Triple Mutation Suppressed Class II CCE in *fugu5* Background. **(A)** Gross and cellular phenotypes. Photographs show the seedling gross morphology, taken at 10 DAS. White bars = 4 mm. Corresponding palisade tissue cells images taken at 25 DAS. Black bars = 50 μm. **(B)** Quantification of cotyledon cellular phenotypes. Cotyledons of each genotype were dissected from plants grown on rockwool for 25 DAS, fixed in FAA, and cleared for microscopic observations. Data show cotyledon cell number, cell areas and cotyledon area. Data are means ± SD (n = 16 cotyledons). Single asterisk indicates that the mutant was statistically significantly different compared to the WT (Tukey’s HSD test at *P* < 0.05; R version 3.5.1), and double asterisk indicates that the quadruple mutant was statistically significantly different compared to *fugu5–1* (Tukey’s HSD test at *P* < 0.05; R version 3.5.1) DAS, days after sowing.

Quantitative analyses revealed that cell numbers, cell sizes and cotyledon sizes in *ibr1-2 fugu5-1*, *ibr3-1 fugu5-1*, and *ibr10-1 fugu5-1* were comparable to the WT (S1 Fig). However, the cotyledon of the *ibr1*,*3*,*10 fugu5-1* quadruple mutant was smaller than that of the WT and *fugu5–1* (Fig 1A and 1B). Subsequent quantification at the cellular level revealed a reduced cell number in *ibr1*,*3*,*10 fugu5–1* to the same extent as in *fugu5–1* and *ibr1*,*3*,*10* (Fig 1B). However, cells in the quadruple mutant were comparable in size to those of the WT (Fig 1B). Note that *fugu5* cotyledon area was not different from the WT (Figs 1B and S1B).

Next we tested whether the reduced cell numbers in *fugu5–1* is due to the lack of Suc, acting as the trigger of CCE [22–25,27]. To do so, we assessed the phenotypic effects of exogenous Suc supply on the *ibr1*,*3*,*10 fugu5–1* quadruple mutant phenotype. While cell numbers in the quadruple mutant cotyledons were reset to the WT levels, the cell size remained unchanged and was comparable to the *fugu5–1* single mutant (S2 Fig). In addition, we noticed smaller rosette leaves, shorter flowering stems in *fugu5–1* and the quadruple mutant compared to the WT and *ibr1*,*3*,*10* (S3 Fig), suggesting a growth delay in *ibr1*,*3*,*10 fugu5–1*, as is seen in the *fugu5–1* single mutant [23,47].

These results indicate that Class II CCE in *fugu5–1* is suppressed when IBA-to-IAA conversion is genetically impaired, mimicking *ech2–1 fugu5–1* [22,25]. Surprisingly, although cell numbers in *ibr1*,*3*,*10* cotyledons were decreased to the same level as in *fugu5–1* (Fig 1B), this phenotype significantly but only partially recovered following exogenous supply of Suc (S2 Fig).

### *ibr10* mutants exhibit Class II CCE

The small cotyledon phenotype in *ibr1*,*3*,*10* has been attributed to the failure of IBA-to-IAA conversion [34]. However, the cellular phenotypes of *ibr1–2*, *ibr3–1*, and *ibr10–1* single mutant cotyledons have not been reported. Although the single mutant gross morphology, and cotyledon aspect-ratios were comparable to that of the WT (S4A and S4B Fig), quantification of their cotyledon cellular phenotypes revealed that while *ibr1–2* and *ibr3–1* display normal cell numbers and cell sizes, *ibr10–1* exhibited significantly fewer cells and thus CCE (S4C Fig).

**Fig 2:**
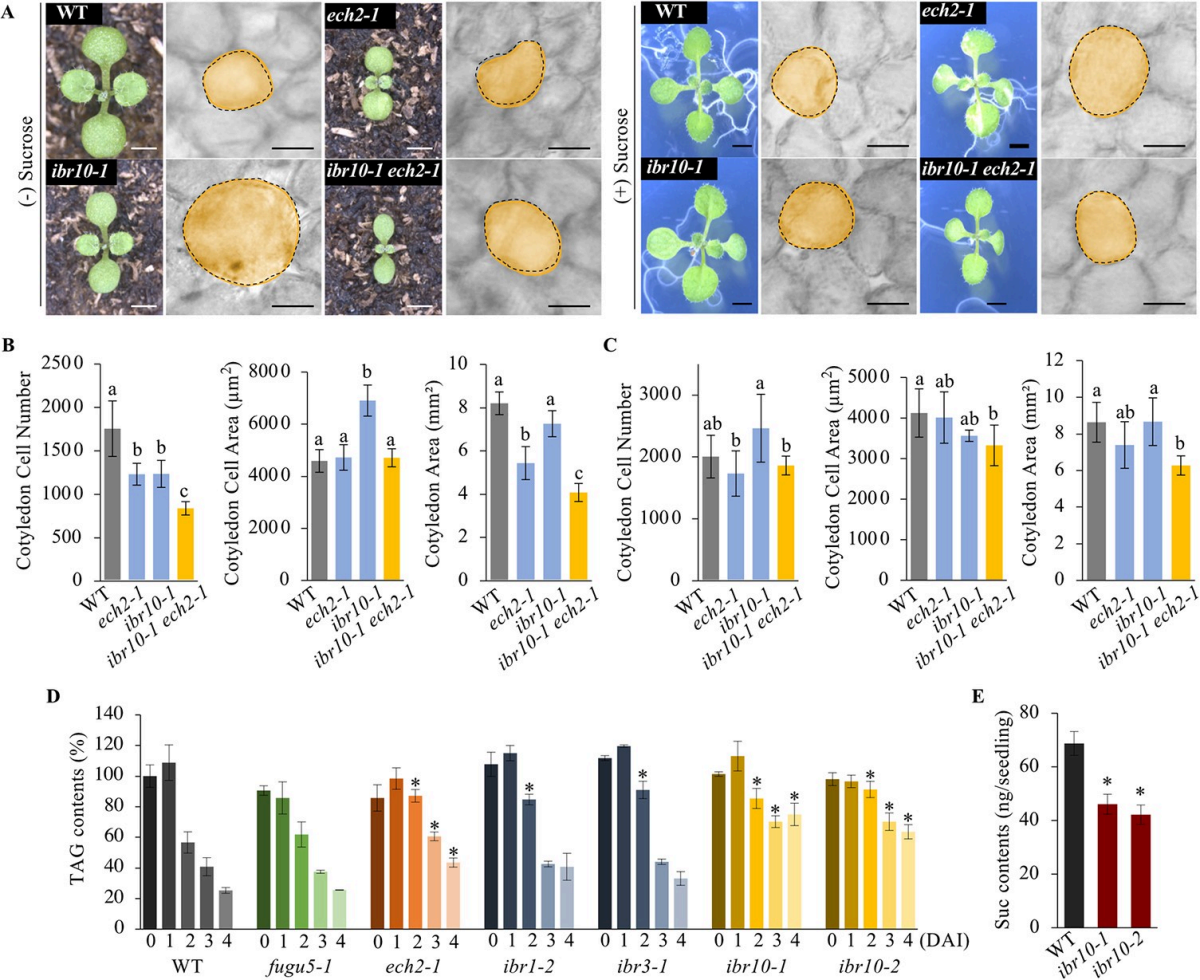
*ibr10* Mutants Exhibit Typical Class II Compensation. **(A)** Seedling gross phenotypes and cotyledon cellular phenotypes. Seedling (left panels) and corresponding cotyledon palisade tissue cells (right panels) of each line grown either on rockwool or MS + 2% Suc medium, respectively. Seedling photographs were taken at 10 DAS. Bar = 5 mm. Palisade tissue cells images were taken at 25 DAS. Bar = 100 μm. DAS, days after sowing. **(B)** Quantification of cotyledon cellular phenotypes. Cell number, cell area and cotyledon area were determined in cotyledons grown on rockwool (without exogenous Suc). Data are means ± SD (n = 8 cotyledons). Letters indicate groups (Tukey’s HSD test at *P* < 0.05; R version 3.5.1). **(C)** Quantification of cotyledon cellular phenotypes. Cell number, cell area and cotyledon area were determined in cotyledons grown on MS + 2% Suc medium for 25 DAS. Data are means ± SD (n = 8 cotyledons). Letters indicate groups (Tukey’s HSD test at *P* < 0.05; R version 3.5.1). **(D)** Time course analyses of TAG breakdown. Seeds of the WT and all mutant lines indicated above were surface-sterilized and sown on MS medium plates without Suc supply. TAG contents were quantified as described in “**Methods**”; 20 dry seeds or 20 etiolated seedlings were used for each measurement. Data are means ± SD (n = three independent experiments; three independent measurements per experiment). Single asterisk represents a statistically significant difference compared to the WT (*P* < 0.01 by Dunnett’s test; R version 3.5.1). DAI, days after induction of seed germination. TAG, triacylglycerol. **(E)** Quantification of Suc contents in etiolated seedlings. 100 etiolated seedlings were collected from MS medium without Suc, and Suc contents were quantified using the internal standard methods of GC-QqQ-MS at 3 DAI. Data are means ± SD (n = five independent experiments; three independent measurements per each experiment). Single asterisk represents a statistically significant difference compared to the WT (*P* < 0.05 by Dunnett’s test; R version 3.5.1).

As the *ech2–1* mutation completely suppresses CCE in *fugu5–1* [22], *ech2–1* was introgressed into *ibr10–1*, and the cellular phenotypes in cotyledons from the double mutant were analyzed. Our results revealed that CCE did not occur in *ibr10–1 ech2–1* despite having a significantly reduced cell number (Fig 2A and 2B). Moreover, exogenous Suc supply cancelled compensation in the *ibr10–1* mutant background (Fig 2A and 2C). Analyses of another allele, *ibr10–2* (SALK_201893C), confirmed our findings (S5A and S5B Fig).

Because cell number can be rescued by the application of exogenous Suc as in the *fugu5* single mutants (Fig 2C; [23]), we next focused on key metabolites in the *ibr10* mutants during seedling establishment. First, quantification of TAG content in dry seeds and etiolated seedlings revealed that although TAG breakdown in *fugu5–1*, *ibr1–2*, and *ibr3–1* single mutants was relatively normal, it was significantly delayed in *ech2–1*, *ibr10–1*, and *ibr10–2* (Fig 2D). Compared to the WT, *fugu5–1* mutants produced less UDP-Glc, and thus less *de novo* Suc due to high cytosolic PPi, but TAG breakdown was almost unaffected [23,27]. However, in the *ibr10* and *ech2–1* mutants, both of which are defective in peroxisomal enzymes, TAG breakdown was markedly impaired (Fig 2D). To gain insight into the effects of the *ibr10* mutations on gluconeogenesis, we quantified the Suc levels. Our measurements revealed that the Suc levels in the *ibr10* mutants were decreased to nearly 60% of the WT (Fig 2E), suggesting that fatty acid β-oxidation was impaired in the *ibr10* mutants. Surprisingly, Suc levels were either slightly increased or unchanged in *ibr1-2* and *ibr3-1*, respectively (S6 Fig). Based on these findings, the *ibr10* mutants could qualify as Class II CCE mutants, with similar properties to *fugu5*. *ibr10–1* was used as a representative allele for the following analyses. Note that CCE in *ibr10–1* was also suppressed in the *ibr1*,*3*,*10–1* triple mutant background (Fig 1B), indicating the importance of IBA-derived IAA for *ibr10–1* cell size increase.

### Defects in IBA efflux further enhance CCE in *fugu5*

IBA is transported over long distances by several carriers, including ABCG36/PEN3/PDR8 [42] and ABCG37/PDR9/PIS1 [42–44], both of which mediate IBA efflux through the plasma membrane (reviewed in [48,49]). These IBA transporters play important regulatory roles in cellular IBA levels (reviewed in [50]). The *pen3–4* mutant displayed high-auxin phenotypes in several organs, including the cotyledons, indicating that cellular IAA levels within these organs are increased due to disrupted IBA outflow [42]. Therefore, we investigated the contribution of *pen3–4* and *pdr9–2* mutations on CCE.

**Fig 3:**
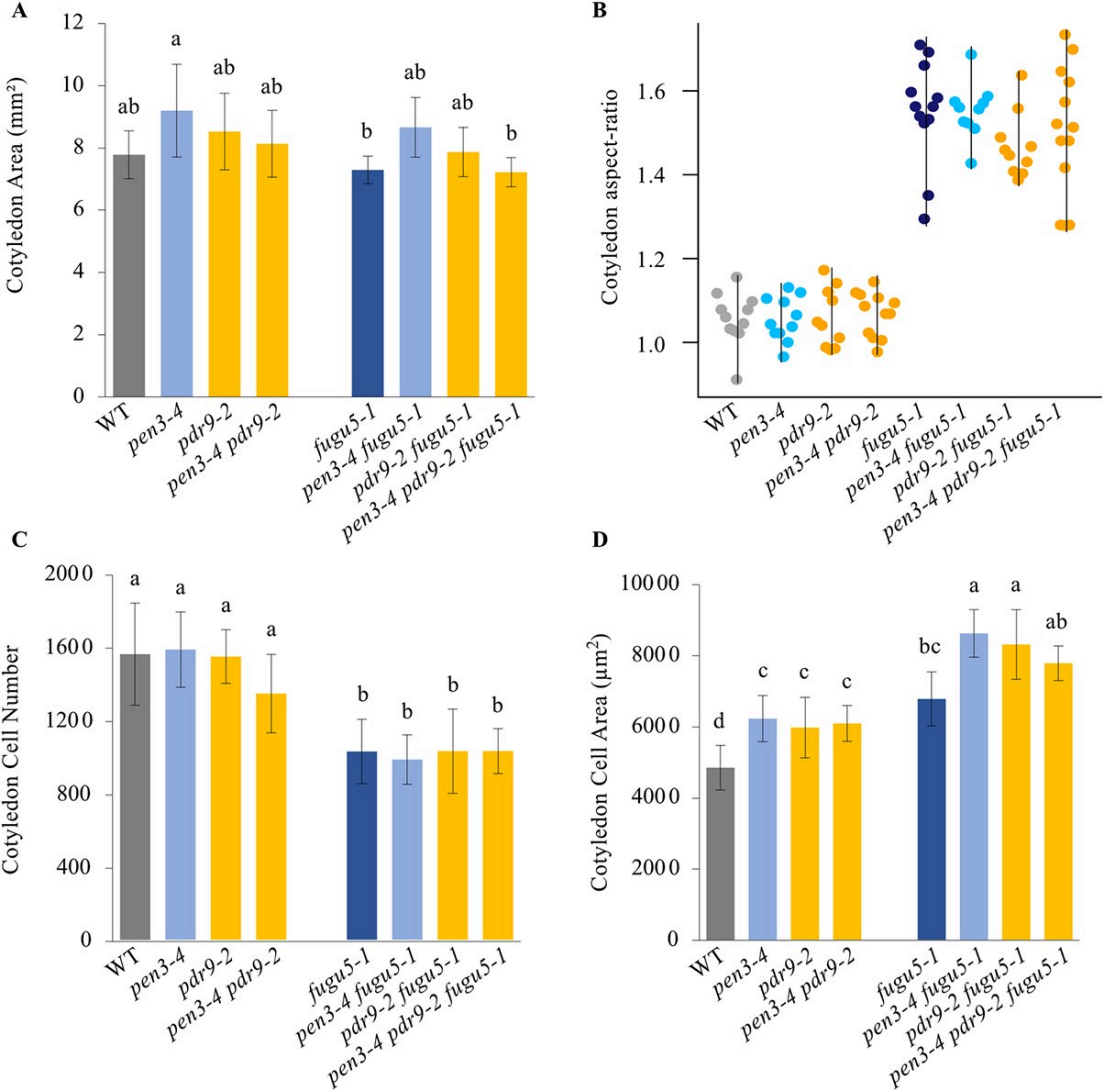
*pen3–4* and *pdr9–2* Mutations Independently and Collectively Enhanced Compensated Cell Enlargement in *fugu5*. Cotyledons were dissected from plants grown on rockwool for 25 DAS, fixed in FAA, and cleared for microscopic observations. Data show the cotyledon area **(A)**, cotyledon aspect-ratio **(B)**, cotyledon cell number **(C)** and cotyledon cell area **(D)**, respectively. Data are means ± SD (n = 8 cotyledons). The cotyledon aspect ratio was calculated by dividing cotyledon blade length by cotyledon blade width. Longer cotyledons have greater aspect-ratio values. Data in the beeswarm plots indicate the value of aspect-ratio (n ≦ 10 cotyledons). Letters indicate groups (Tukey’s HSD test at *P* < 0.05; R version 3.5.1). DAS, days after sowing.

To examine the effects of IBA accumulation on CCE, we generated *pen3–4 fugu5–1*, *pdr9–2 fugu5–1*, and *pen3–4 pdr9–2 fugu5–1* mutant combinations and compared their cotyledon cellular phenotypes to *fugu5–1*. All double and triple mutants in the *fugu5–1* background exhibited slightly bigger oblong cotyledons, reminiscent of *fugu5–1*, as indicated by the cotyledon size (Fig 3A) and cotyledon aspect-ratios (Fig 3B). On the other hand, while cell numbers in *pen3–4*, *pdr9–2*, and *pen3–4 pdr9–2* were almost unaffected (Fig 3C), cell sizes were significantly larger than the WT (Fig 3D). Notably, cells in *pen3–4 fugu5–1*, *pdr9–2 fugu5–1* were significantly larger than those in *fugu5–1* single mutants (Fig 3D), indicating that CCE was enhanced due to the high IBA levels. In other words, the *pen3–4* and *pdr9–2* mutations promoted CCE. Hereafter, this phenotype will be referred to as overcompensated cell enlargement (OCE).

### Role of the ARF-mediated auxin response in CCE

Based on the genetic evidence, CCE in *fugu5* can either be enhanced or suppressed in an IBA-derived IAA-level dependent manner. In general, IAA signaling is mediated by AUX/IAA proteins and ARF transcription factors (TFs) [51–55]. In this study, we focused on ARF7 and ARF19 because they are strongly expressed in cotyledons from 1-week-old seedlings [56]. To address the role of IAA signaling in *fugu5*, we generated *arf7–1 fugu5–1*, *arf19–1 fugu5–1* and *arf7–1 arf19–1 fugu5–1* mutants and analyzed their cellular phenotypes.

**Fig 4:**
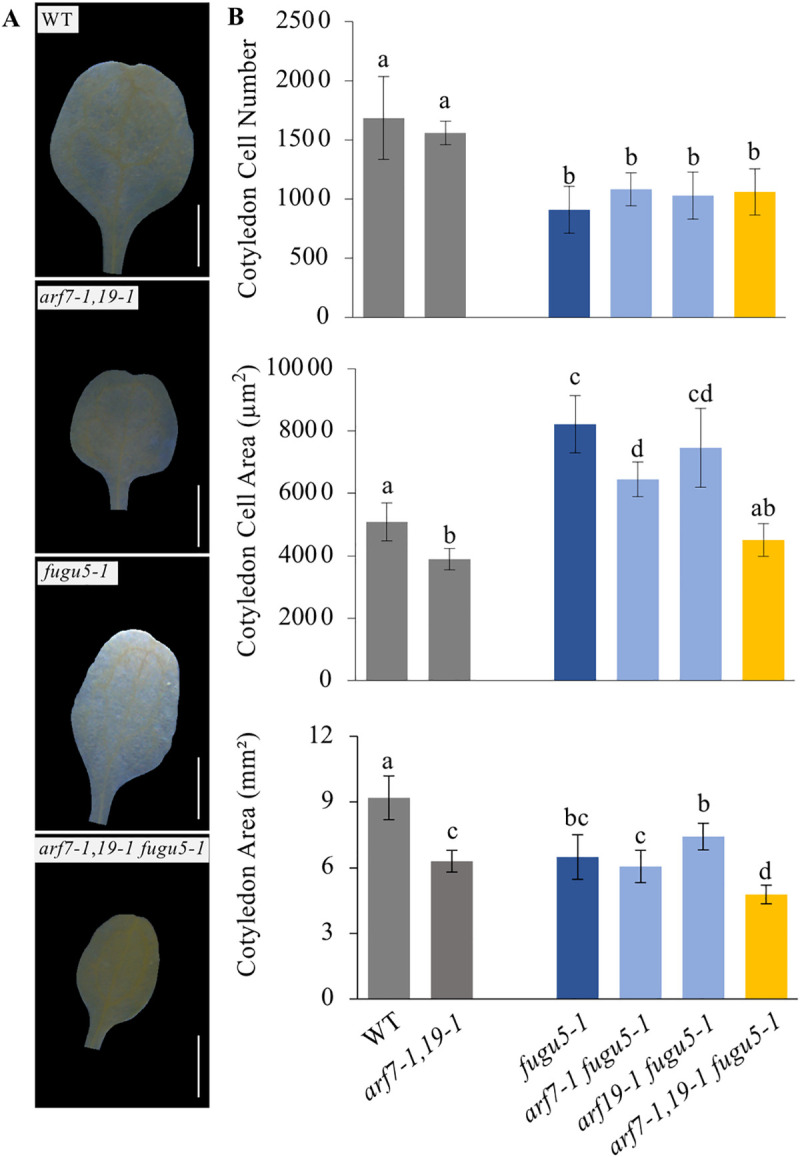
*arf7–1 arf19–1* Mutations Suppressed Compensated Cell Enlargement in the *fugu5* Background. **(A)** Effects of the *arf7–1* and *arf19–1* mutations on morphological and cellular phenotypes. Photographs show the gross morphology of the WT, *fugu5–1*, *arf7–1 arf19–1*, and *arf7–1 arf19–1 fugu5–1* (*arf7-1*, *19-1 fugu5-1*) cotyledons at 25 DAS. White bars = 1 mm. DAS, days after sowing. **(B)** Quantification of cotyledon cellular phenotypes. Cotyledons of the above genotypes were dissected from plants grown on rockwool for 25 DAS, fixed in FAA, and cleared for microscopic observations. Data represent cotyledon cell numbers, cell areas and cotyledon areas. Data are means ± SD (n = 8 cotyledons). Letters indicate groups (Tukey’s HSD test at *P* < 0.03; R version 3.5.1).

Our results indicated that the cotyledon size in *arf7–1 arf19–1* and *arf7–1 arf19–1 fugu5–1* mutants was notably smaller when compared to WT or *fugu5–1* (Fig 4A and 4B). Next, quantification of cotyledon cellular phenotypes revealed that all of the mutants in the *fugu5–1* background displayed significantly decreased cotyledon cell numbers (Fig 4B). In addition, the cotyledonary palisade tissue cells in the *arf7–1 fugu5–1* double mutant were slightly smaller than those in *fugu5–1* (Fig 4B). Importantly, cell size in *arf7–1 arf19–1 fugu5–1* was indistinguishable from the WT (Fig 4B). These findings indicate that an auxin signaling pathway involving both ARF7 and ARF19 might play a major role in driving CCE in *fugu5–1*.

### High endogenous concentration of IAA drives CCE in *fugu5*

**Fig 5:**
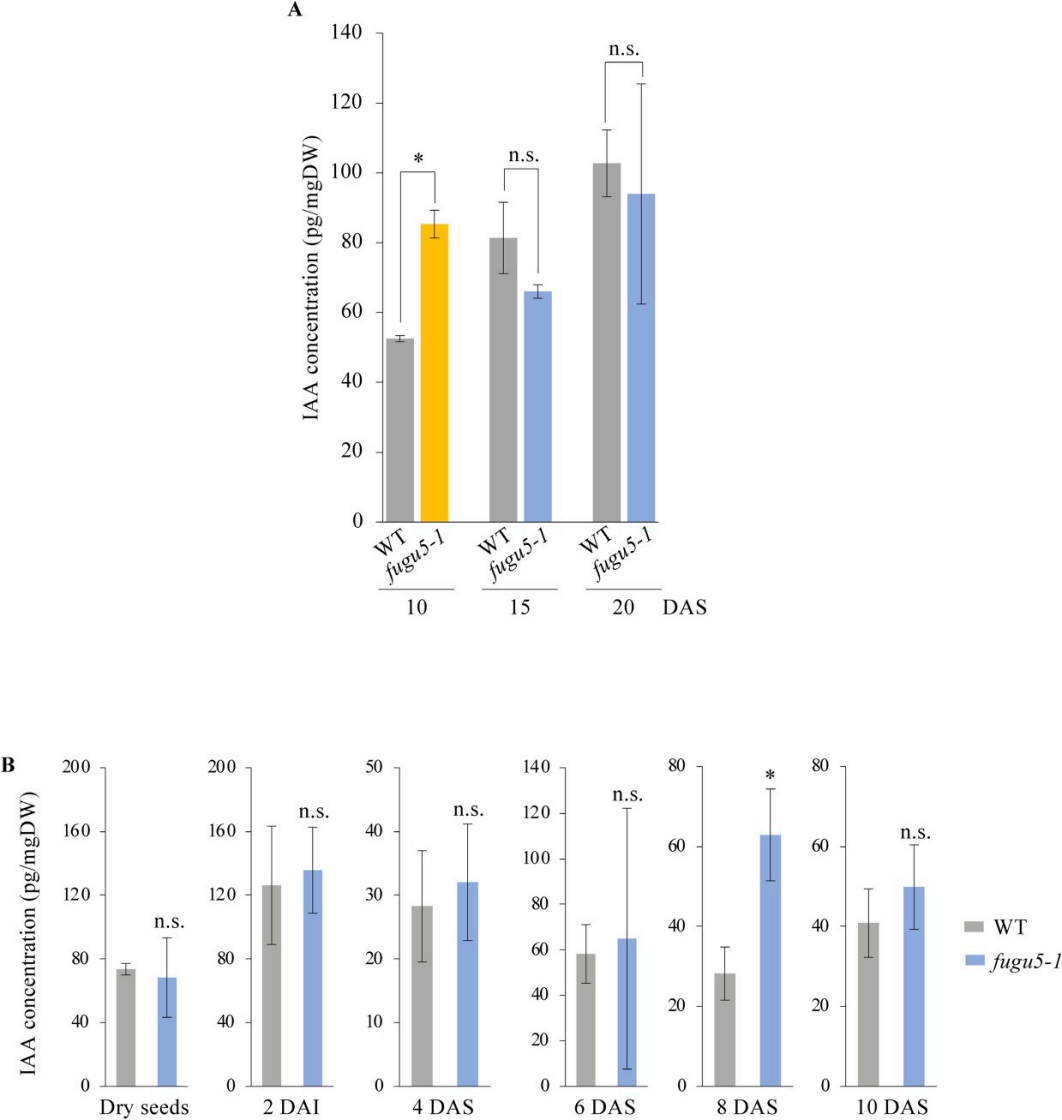
Endogenous Concentration of IAA in *fugu5–1* Shoots at Several Developmental Stages. **(A)** Quantification of endogenous IAA at different developmental stages. Cotyledons of the WT and *fugu5–1* were collected at 10, 15, and 20 DAS. Data are means ± SD (n = 3 independent experiments). Single asterisk indicates that *fugu5–1* was statistically significantly different compared to the WT (Student’s t-test at *P* < 0.05). NS, not significant. DAS, days after sowing. **(B)** Time-course quantification of endogenous IAA in the WT and *fugu5–1*. Samples consisted of dry seeds or whole seedling shoots collected at 2 DAI, or 4, 6, 8 and 10 DAS. Data are means ± SD (n ≥ 3 independent experiments). Single asterisk indicates that *fugu5–1* was statistically significantly different compared to the WT (Student’s t-test at *P* < 0.05). DAI, days after induction of seed germination.

Our results strongly suggest that IAA was the key molecule driving Class II CCE in *fugu5*. In addition, previous kinematic analyses had revealed that cell expansion peaks in *fugu5–1* cotyledons around 15 days after sowing (DAS) [3]. To relate both findings, IAA concentration was quantified by a UPLC-Q-TOF-MS system using cotyledons dissected from seedlings at 10, 15 and 20 DAS. We found that the IAA concentration was 1.7-fold higher in *fugu5–1* than in the WT at 10 DAS (Fig 5A). At 15 and 20 DAS, IAA concentration was not significantly different among *fugu5–1* and the WT anymore (Fig 5A). Next, we quantified IAA in a time-course manner in dry seeds, or in young seedling shoots collected at 2 days after induction of seed germination (DAI), and at 4, 6, 8, and 10 DAS. There was no significant difference in IAA concentration between the WT and *fugu5–1* in the dry seeds and seedlings collected at 2 DAI, 4 and 6 DAS (Fig 5B). Surprisingly, at 8 DAS, *fugu5–1* seedlings contained 2.2-fold more IAA that the WT (Fig 5B).

To validate the above results, IAA concentration was further quantified in higher-order mutant combinations, namely *ibr1*,*3*,*10–1*, *ibr1*,*3*,*10–1 fugu5–1*, and *ibr1*,*3*,*10–1 pen3-4 fugu5–1*, in which CCE is suppressed (Figs 1, S7A and S7B). We found that IAA levels in all the above higher-order mutant lines were comparable to the WT (S8 Fig). This suggests that the accumulation of IAA in cotyledons which occurs in *fugu5–1* at 8–10 DAS (Fig 5A and 5B) is a key transition point to drive CCE in *fugu5* cotyledons.

### IBA-to-IAA conversion is the driving force for CCE in all Class II compensation-exhibiting mutants

Consistent with its suppressive effect on *fugu5* CCE [22], the *ech2–1* mutation completely suppressed CCE in *icl–2*, *mls–2*, *pck1–2* [25] and *ibr10* (Figs 2 and S5).

**Fig 6:**
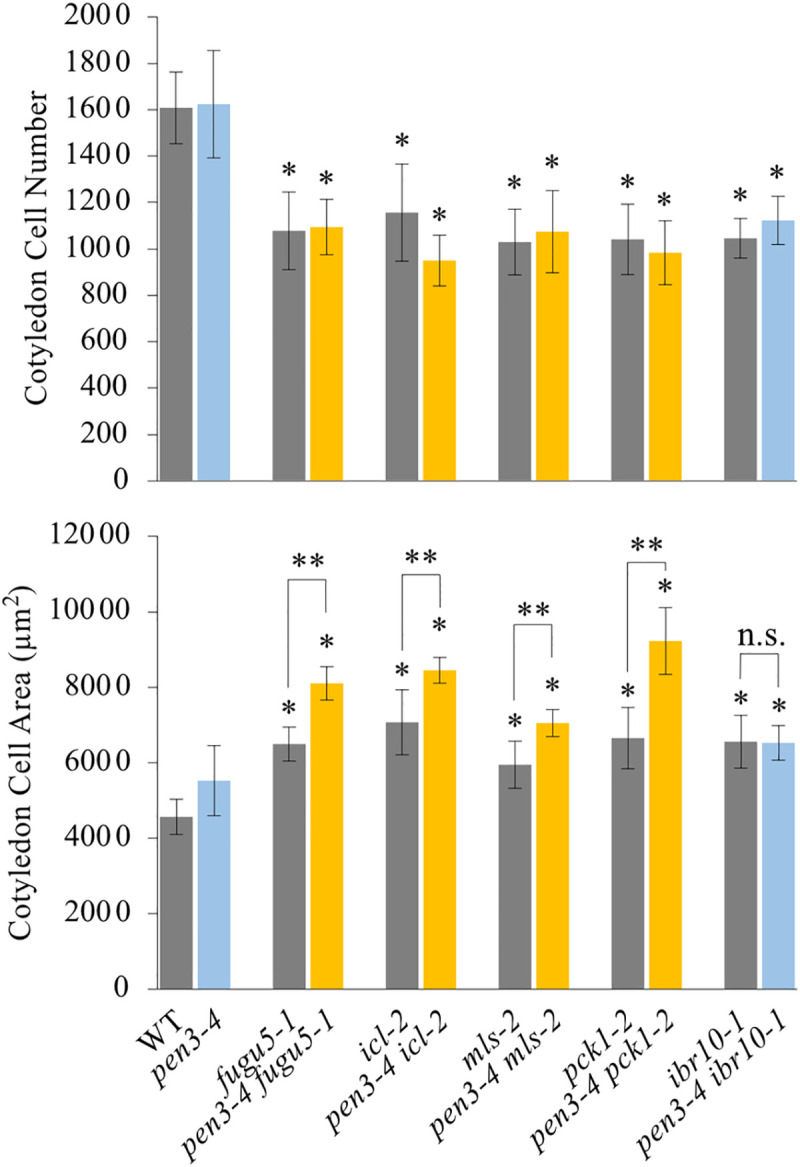
Effect of Increased IBA on Compensated Cell Enlargement in Class II Mutants. Plants were grown on rockwool for 25 DAS; their cotyledons were dissected, fixed in FAA, and cleared for microscopic observations. Data represent cotyledon cell numbers and cotyledon cell areas. Data are means ± SD (n = 8 cotyledons). Single asterisk indicates that the mutant was statistically significantly different compared to the WT (*P* < 0.05 by Tukey’s HSD test; R version 3.5.1). Double asterisk indicates that single mutants were statistically significantly different compared to the corresponding double mutants in the *pen3–4* background (*P* < 0.05 by Tukey’s HSD test; R version 3.5.1). DAS, days after sowing.

Next, to validate the role of IBA in Class II CCE, we constructed double mutants between the above mutants and *pen3–4*. Our results indicate that while the *pen3–4* mutation triggered OCE in *icl–2*, *mls–2*, and *pck1–2*, in *ibr10–1* CCE remained unaffected in the *pen3–4* background (Fig 6). Taken together, these results further confirm that Class II CCE is promoted by IBA-derived IAA.

### Vacuole acidification *via* the V-ATPase activity is essential for CCE in *fugu5*

**Fig 7:**
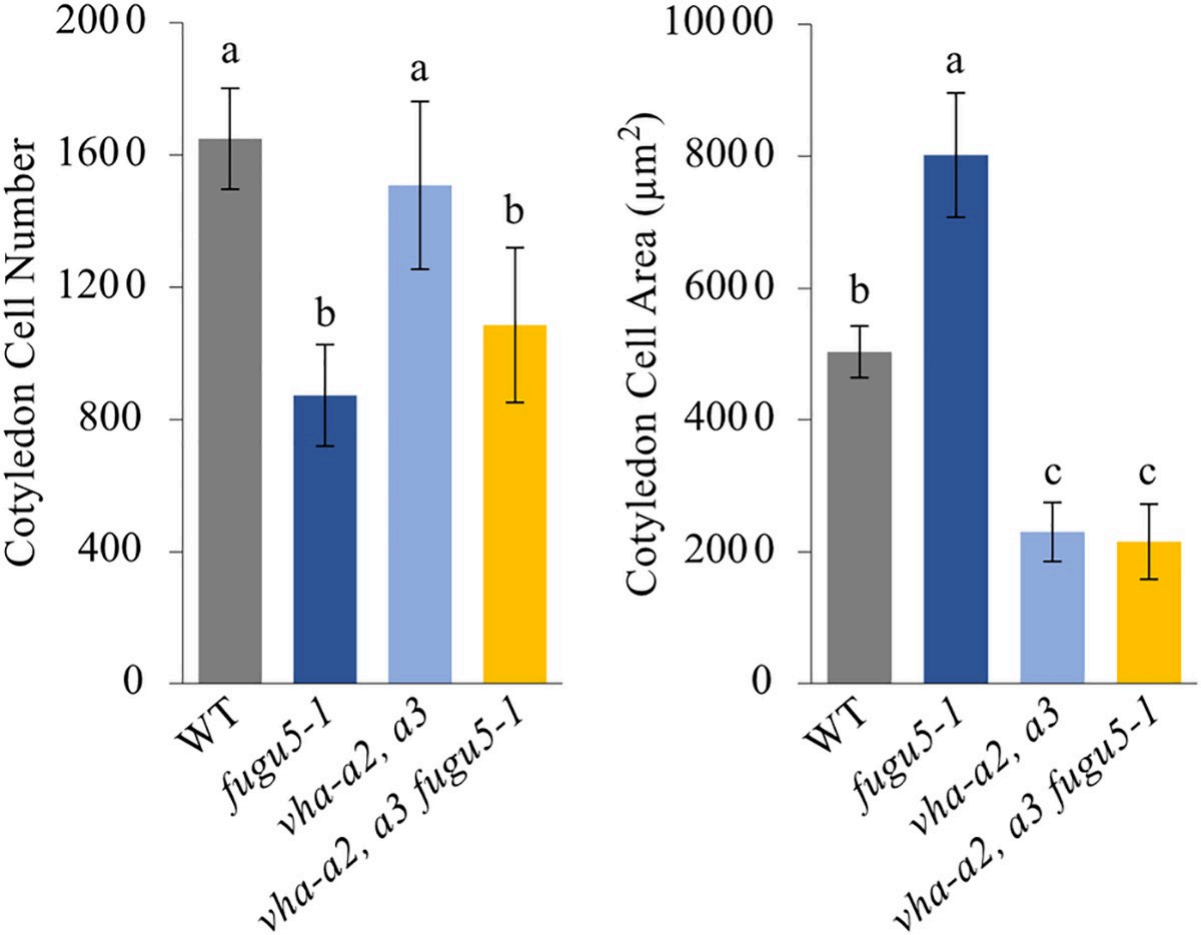
V-ATPase Complex Activity is Essential for Compensated Cell Enlargement in *fugu5*. Effect of suppressed V-ATPase activity on CCE. Cotyledons of the WT, *fugu5–1*, and *vha-a2 vha-a3* (*vha-a2*, *a3*) double mutants, and *vha-a2 vha-a3 fugu5-1* (*vha-a2*, *a3 fugu5-1*) triple mutants were dissected from plants grown on rockwool for 25 DAS, fixed in FAA, and cleared for microscopic observations. Data represent cotyledon cell numbers and cotyledon cell areas. Data are means ± SD (n = 8 cotyledons). Letters indicate groups (Tukey’s HSD test at *P* < 0.05; R version 3.5.1). DAS, days after sowing.

Vacuoles are not only the largest organelles of mature plant cells, they also play pivotal roles in plant growth and development, notably through the regulation of turgor pressure. This might involve vacuole acidification through the V-ATPase, downstream of auxin signaling [45,46]. To investigate the putative contribution of V-ATPase-dependent vacuole acidification on *fugu5* CCE, we analyzed the *vha-a2 vha-a3 fugu5–1* triple mutant. Note that the *vha-a2 vha-a3 fugu5–1* triple mutant has no H^+^-pumping activity across the tonoplast, resulting in nearly null ΔpH [57]. Strikingly, the *vha-a2 vha-a3* [57,58], which harbors mutations in two a-subunits of the V-ATPase, completely suppressed CCE in the *fugu5–1* background (Fig 7). This suggests that the H^+^ pumping activity *via* the V-ATPase complex is essential for CCE.

## Discussion

### Coupling between cell proliferation and cell expansion during leaf morphogenesis

A longstanding question in biology is how organ size is determined [4,10]. How do organs know when to stop growing? Is size merely a function of the proliferative growth of individual cells, or is it rather determined by a global control system that acts at the level of the whole organ?

In the simplest scenario, leaf size would be a function of cell number and size. However accumulating evidence suggests that a severe decrease in cell number usually triggers excessive cell enlargement post-mitotically. The inverse relationship that exists between the two major determinants of leaf size, namely cell number and size, was first described by the so-called “compensation” [9]. In other words, compensation highlights the coupling between cell division and expansion at the level of the entire organ. Over the last few years, the number of compensation-exhibiting mutants has increased, suggesting that compensation might reflect a general size regulatory mechanism in plants. Here we focus on the *fugu5* and report how cell expansion can compensate for lower cell division during morphogenesis, whereby IBA-to-IAA conversion plays a key role, shedding light on the mechanisms coupling these processes during organ development.

### IBA-derived IAA is the driving factor of CCE in *fugu5*

Compensation consists of two interdependent stages: the induction stage and the response stage [16]. While the induction stage in *fugu5* (i.e., the mechanism leading to the reduction in cell number) has been extensively investigated, the response stage mechanism that mediates CCE remains largely unknown. However, two studies have provided some insights into this stage. In the first study, genetic screening and phenotypic analyses revealed that CCE in *fugu5* cotyledons was specifically suppressed by *ech2–1* mutations. Therefore, we proposed that CCE in *fugu5* is driven by IBA-derived IAA [22]. The second study validated the above hypothesis and further demonstrated that for Class II CCE to occur, the peroxisomal IBA-to-IAA conversion was a prerequisite not only in *fugu5* but also in *icl–2*, *mls–2*, and *pck1–2* [25]. In this study, we analyzed the contribution of the phytohormone auxin behind CCE by generating higher-order mutant lines where endogenous level of IBA was specifically up- or downregulated. We also quantified the endogenous IAA levels in *fugu5*, analyzed the role of auxin signaling in this process by mutating key TFs (ARF7 and ARF19), and opening to the potential implication of the vacuole in CCE.

Collectively, our results revealed that while CCE was completely suppressed in *ibr1*,*3*,*10–1 fugu5–1*, it was significantly enhanced in *pen3–4 fugu5–1* and *pdr9–2 fugu5–1*, resulting in OCE (Figs 1 and 3). Thus, it became evident that IBA-to-IAA conversion is a prerequisite for CCE to occur in *fugu5–1*.

Interestingly, the average cell size in *ibr1*,*3*,*10–1 pen3–4 fugu5–1* was similar to that of the WT (S7 Fig). The *ibr1*,*3*,*10–1 pen3–4* also displayed the low auxin phenotype, similar to that of *ibr1*,*3*,*10–1*. This occurred because additional IBA accumulation due to the *pen3–4* mutation was not properly converted into IAA due to the *ibr1*,*3*,*10–1* mutations [34]. The OCE observed in *pen3–4 fugu5–1* was completely suppressed in the *ibr1*,*3*,*10–1 pen3–4 fugu5–1* quintuple mutant, in which the cell size was reset to the WT levels (S7B Fig). Consistently, the cell size of the different *ibr pen3–4 fugu5–1* triple mutant combinations were comparable to that of *fugu5–1* (S9 Fig). Therefore, CCE in *fugu5–1* is either suppressed or enhanced in an IAA concentration-dependent manner. Although IBA is not recognized by the auxin TIR1/AFBs receptor [59,60], it represents a precursor for IAA biosynthesis. Therefore, by serving as a source of auxin, IBA plays a major role in IAA supply sustaining CCE during *fugu5* cotyledon development.

### An IAA/ARF signaling pathway transduces signals that trigger CCE in *fugu5*

Auxin signaling regulates various developmental processes, such as cell division, cell growth, or cell differentiation *via* AUX/IAA proteins and ARF TFs. Previous studies have shown that ARF7 and ARF19 promote lateral and adventitious root formation [55,56], and redundantly promote leaf cell expansion [56].

To clarify the relationship between ARF7, ARF19, IAA and CCE, we used the *arf7–1 arf19–1 fugu5–1* triple mutant because, as mentioned above, ARF7 and ARF19 are strongly expressed in cotyledons of 1-week-old seedlings and are involved in cell expansion control [56]. Cellular phenotypic analyses of the cotyledons of the above triple mutants revealed that only cell size was decreased, but cell numbers were unchanged (Fig 4B), indicating that CCE in *fugu5–1* was completely suppressed by the *arf7–1 arf19–1* mutations (Fig 4). Altogether, these findings indicate that the CCE in *fugu5–1* cotyledons was triggered by IAA *via* the ARF7 and ARF19 TFs related signaling pathways.

The above findings were further supported by the increase in endogenous IAA concentration in *fugu5–1*, which was significantly higher than the WT, particularly at 8–10 DAS (Fig 5), the stage during which post-mitotic expansion is exponentially increased (see kinematic analyses in [3]). While IAA concentrations in *fugu5* were trending upward (Figs 5A and 5B and S8), the differences in IAA concentration between the WT, *fugu5-1* and higher order mutant lines with suppressed CCE were not statistically significant (S8 Fig). This discrepancy in IAA concentrations could be due to slight differences in plant growth between independent experiments, and/or to the homeostasis. In fact, as mentioned above, besides its highly mobile nature, endogenous IAA levels are maintained through complex, yet specific metabolic pathways that can release/store IAA from/into its inactive forms, or synthesize IAA *de novo* from several independent pathways [35–37,61]. Hence, although measuring such low hormone levels has been challenging, the variability inherent among independent experiments and different genetic backgrounds should be considered carefully.

Finally, vacuolar acidification mediated by V-ATPase complex has been shown to play a crucial role in *fugu5* CCE (Fig 7), which corresponds with the role of turgor pressure in distended vacuoles during post-mitotic cell expansion [62–64]. According to the so-called “acid growth theory”, it is widely accepted that IAA activates the plasma membrane H^+^-ATPase, acidifies the apoplast and activates a range of enzymes involved in cell wall loosening ([65]; for a review see [66]). Although the above theory links IAA-mediated extracellular acidification and subsequent cell wall loosening to turgor-dependent cell expansion [67], little is known about the role of the tonoplast in the IAA-mediated growth of plant cells. Importantly, increased vacuolar occupancy has been recently shown to allow cell expansion through a mechanism that requires LRXs and FER module, which senses and conveys extracellular signals to the cell to ultimately coordinate the onset of cell wall acidification and loosening with the increase in vacuolar size [68]. More recently, it has been reported that plant vacuoles significantly increase their volume (ca. two-fold) upon incubation with 1 μM IAA [45,46], linking auxin and vacuolar acidification, and providing insights into vacuole volume regulation during post-mitotic plant cell expansion.

**Fig 8:**
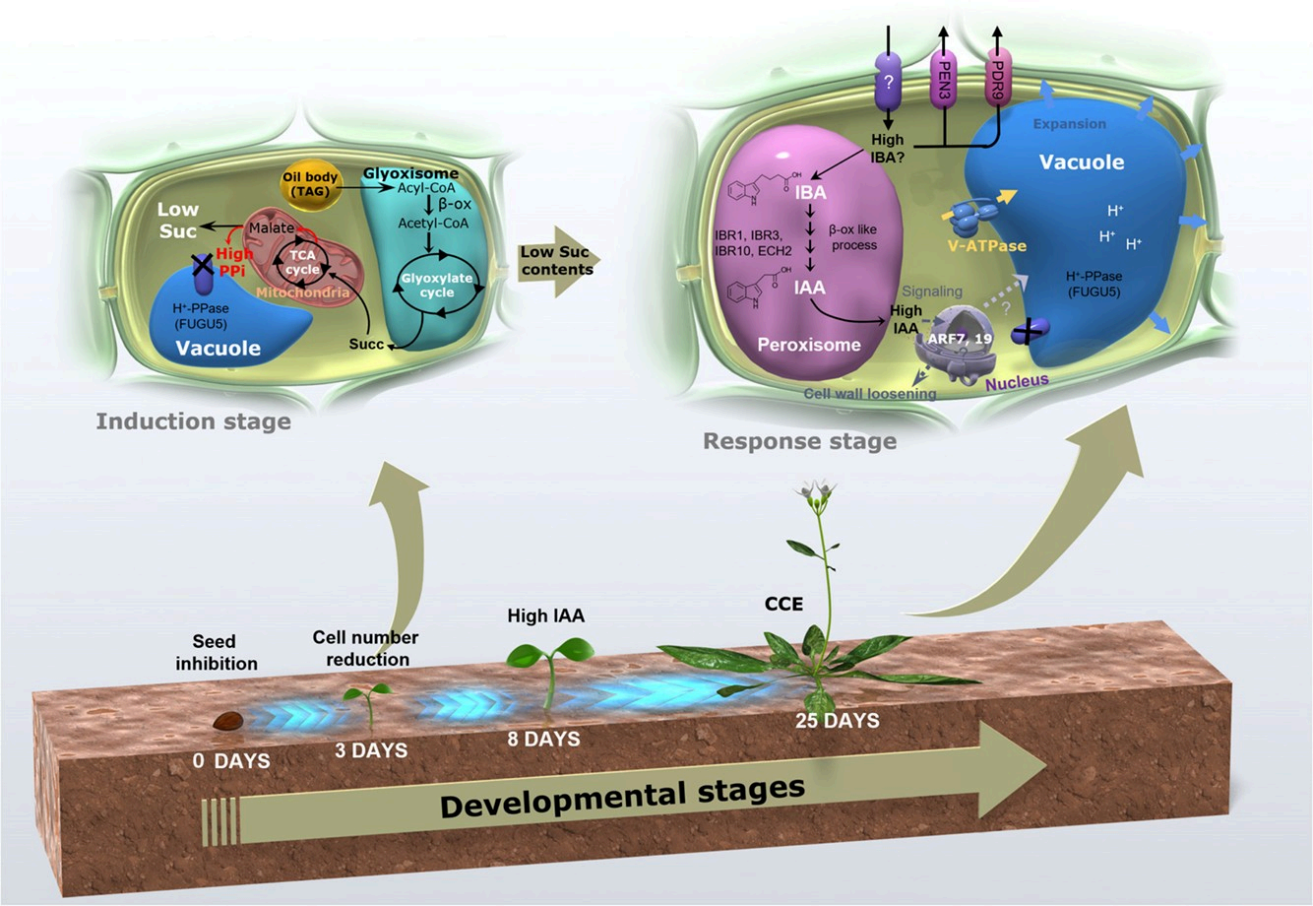
Proposed Model for Class II CCE. We previously reported a working model for Class II CCE [25], showing that decreased cell numbers in cotyledons were exclusively attributed to decreased TAG-derived Suc, and that CCE might be mediated by IBA-derived IAA related mechanism. Here, we confirmed that IBA-derived IAA is essential for enhancing post-mitotic cell expansion. Based on our previous findings and this research, the scenario for Class II CCE in *fugu5* can be summarized as follows. First, upon seed imbibition, excess cytosolic PPi in *fugu5–1* leads to inhibition of Suc synthesis *de novo* from TAG, by inhibiting the gluconeogenic cytosolic enzyme UDP-glucose pyrophosphorylase (UGPase; [27]). Second, during seedling establishment, reduced Suc contents somehow promote the IBA-to-IAA conversion and lead to increased endogenous IAA concentration at 8–10 DAS, which is apparently a crucial transition point to drive CCE in *fugu5* cotyledons (Fig 5). Third, one can assume that high endogenous IAA triggers the TIR/AFB-dependent auxin signaling pathway through ARF7 and ARF19, and subsequently activates the vacuolar type V-ATPase leading to an increase in turgor pressure. This ultimately triggers cell size increase and CCE. This scenario is also valid for other mutants, namely *icl-2*, *mls-2*, *pck1–2*, and *ibr10–1*, all of which exhibit a typical Class II CCE, not because of excess PPi, but due to compromised gluconeogenesis from TAG [25]. Finally, this IAA-mediated CCE is not valid for Class I [22]. Nonetheless, our findings that V-ATPase activity is critically important for CCE in Class II and Class III [20, 21], may suggest that all three CCE classes may converge at this checkpoint and use the V-ATPase complex activity as the final driving force to inflate cell size. Although the above scenario is plausible, its robustness still needs to be challenged experimentally in the future. Succ, succinate. DAS, days after sowing.

Based on the above results, the major events involved in the compensation process in *fugu5* can be summarized as shown in our working model (Fig 8). First, during germination, excess cytosolic PPi inhibits Suc synthesis *de novo* from TAG, causing a significant decrease in the number of cells in *fugu5* cotyledons [23]. Second, the metabolic reaction of IBA-to-IAA conversion is somehow promoted, resulting in increased IAA concentration at 8–10 DAS (Fig 5A and 5B), which seems to be a crucial transition point to drive CCE in *fugu5* cotyledons. Third, one can assume that high endogenous IAA triggers the TIR/AFB-dependent auxin signaling pathway through ARF7 and ARF19, and subsequently activates the vacuolar type V-ATPase leading to an increase in turgor pressure. This ultimately triggers cell size increase and CCE (Fig 8). Although the above scenario is plausible, its robustness still needs to be challenged experimentally in the future.

### *Ibr10* is a new Class II compensation exhibiting mutant

Our work links CCE, auxin and metabolism. Indeed, mobilization of TAG is essential for *Arabidopsis* seed germination and seedling establishment [26,69]. Because *ibr10* etiolated seedlings were as long as the WT, IBR10 was thought not to be involved in the degradation of seed stored lipids [34,39]. However, our analyses focusing on cotyledons revealed that both *ibr10* mutant alleles displayed slow FAs degradation (Fig 2D), produced less Suc *de novo* from TAG (Fig 2E), and exhibited CCE (Fig 2B). On the contrary, the fact that *ibr1–2* and *ibr3–1* have no defects in TAG degradation and Suc synthesis, may suggest that they are functionally different from *ibr10* with respect to TAG mobilization (Figs 2D and S6). On the other hand, our results indicated that while CCE was not suppressed in *ibr1-2 ibr3-1 fugu5-1*, *ibr1-2 ibr10-1 fugu5-1*, and *ibr3-1 ibr10-1 fugu5-1*, it was totally suppressed only in *ibr1*,*3*,*10 fugu5-1* quadruple mutants (Figs 1B and S7B). Altogether, the above results suggest that the loss of function of all possible *ibr* double mutant combinations in *fugu5* background are not sufficient to suppress CCE, and point to their redundant enzymatic role in IBA-to-IAA conversion. Although it remains unclear how IBR10 contributes to FAs degradation, our findings suggest that IBR10 plays a pivotal role during stored lipid mobilization in *Arabidopsis*.

The *icl–2*, *mls–2*, and *pck1–2* mutants, which have a metabolic disorder in the TAG to Suc pathway, displayed short etiolated seedling phenotypes [25]. Although *pck1–2* has the lowest Suc contents, we previously reported that hypocotyl elongation of *pck1–2* etiolated seedlings was only mildly affected compared to *icl–2* and *mls–2* [25]. Classically, the elongation defects in etiolated seedlings of *Arabidopsis* have been ascribed to a deficit in endogenous Suc during seedling establishment [26]. However, our findings confirmed that the length of etiolated seedling does not necessarily reflect Suc availability, and that hypocotyl elongation in the dark is a more complex trait than was expected.

### Low sucrose contents promote the increase in endogenous IAA levels

As described above, the *ech2–1* mutation completely suppressed CCE in *icl–2 mls–2*, *pck1–2*, and *ibr10–1* (Fig 2B; [25]). However, CCE in Class I mutants was not suppressed by the *ech2–1* mutation [22]. These findings suggest that IBA-to-IAA conversion is involved explicitly in driving Class II CCE. Each of the Class II CCE mutants has a metabolic disorder in the TAG-to-Suc pathway during the early stages of post-germinative development. Therefore, the low-sugar-state in seedlings somehow resulted in metabolic changes in the IBA-related metabolism. In brief, Class II compensation is fully controlled by metabolic networks. Indeed, Suc produced by photosynthesis is converted into phosphoenolpyruvic acid (PEP), which in *Arabidopsis*, is subsequently converted into tyrosine, phenylalanine, and tryptophan by the shikimic acid pathway [70]. On the other hand, IAA is a metabolite synthesized from the aromatic amino acid tryptophan [71]. This suggests that the gluconeogenesis pathway and the IAA biosynthesis pathway are tightly related. To this end, it would be interesting to revisit the shade avoidance response mechanism based on the above findings because the low-sugar-state under a low-light regime might trigger IBA-to-IAA conversion and concomitant cell elongation.

Living organisms produce thousands of metabolites at varying concentrations and distributions, which underlies the complex nature of metabolic networks. In this study, several key metabolites were identified to play a role in cell/cotyledon size control. Our findings provide the basis for further research to elucidate the mechanisms of organ-size regulation in multicellular organisms. More specifically, while organ-size regulation has been interpreted by specific sets of key TFs over the last decades, applying metabolomics might be a useful approach in efforts aiming to identify metabolites playing key roles in organ-size control in other kingdoms.

## Methods

### Plant materials and growth conditions

The WT plant used in this study was Columbia-0 (Col-0), and all of the other mutants were based on the Col-0 background. Seeds of *ibr1–2*, *ibr3–1*, *ibr10–1*, *pen3–4*, and *ech2–1* were a gift from Professor Bonnie Bartel (Rice University). *icl–2*, *mls–2*, and *pck1–2* mutants seeds were a gift from Professor Ian Graham (The University of York).

Seeds were sown on rockwool (Nippon Rockwool Corporation), watered daily with 0.5 g L^-1^ Hyponex solution and grown under a 16/8 h light/dark cycle with white light from fluorescent lamps at approximately 50 μmol m^-2^ s^-1^ at 22°C.

Sterilized seeds were sown on MS medium (Wako Pure Chemical) or on MS medium with 2% (w/v) Suc where indicated, and solidified using 0.2–0.4% (w/v) gellan gum to determine the effects of medium composition on plant phenotype. After sowing the seeds, the MS plates were stored at 4°C in the dark for 3 d. After cold treatment, the seedlings were grown either in the light (for the cellular phenotype analyses) or in the dark (for Suc or TAG quantification) for the designated periods of time.

### Mutant genotyping and higher-order mutant generation

*fugu5–1* was characterized as the loss-of-function mutant of the vacuolar type H^+^-PPase [23]. *fugu5–1*, *icl–2*, *mls–2*, *pck1–2*, *ech2–1*, *ibr1–2*, *ibr3–1*, *ibr10–1*, and *pen3–4* were genotyped as described previously (S1 Table; [25,28–30,33]). *ibr10–2* and *pdr9–2* were obtained from the *Arabidopsis* Biological Resource Center (ABRC/The Ohio State University) and genotyped using specific primer sets (S1 Table). *fugu5–1* plants were crossed with other mutants to obtain double, triple, quadruple or quintuple mutants, and the genotypes of the higher-order mutants were checked in the F_2_ plants using a combination of PCR-based markers.

### Microscopy

Photographs of the gross plant phenotypes at 10 DAS were taken with a stereoscopic microscope (M165FC; Leica Microsystems) connected to a CCD camera (DFC300FX; Leica Microsystems), and those at 25 DAS were taken with a digital camera (D5000 Nikkor lens AF-S Micro Nikkor 60 mm; Nikon).

Cotyledons were fixed in formalin/acetic acid/alcohol and cleared with chloral solution (200 g chloral hydrate, 20 g glycerol, and 50 mL deionized water) to measure cotyledon areas and cell numbers, as described previously [72]. Whole cotyledons were observed using a stereoscopic microscope equipped with a CCD camera. Cotyledon palisade tissue cells were observed and photographed under a light microscope (DM-2500; Leica Microsystems) equipped with Nomarski differential interference contrast optics and a CCD camera. Cell size was determined as the mean palisade cell area, detected from a paravermal view, as described previously [23]. The cotyledon aspect ratio was calculated as the ratio of the cotyledon blade length to width.

### Quantitative analyses of total TAGs

The quantities of seed lipid reserves in dry seeds and in 1-, 2-, 3-, and 4-day-old etiolated seedlings were measured by determining the total TAG using the Triglyceride E-Test assay kit (Wako Pure Chemicals). Either 20 dry seeds or 20 seedlings were homogenized with a mortar and pestle in 100 μL sterile distilled water. The homogenates were mixed with 0.75 mL reaction buffer provided in the kit, as described previously [23,73]. The sample TAG concentration was determined according to the manufacturer’s protocol. The length of the etiolated seedlings was determined as described previously [23].

### Quantification of sucrose

Etiolated seedlings at 3 DAI were collected in one tube in liquid nitrogen and were freeze-dried. Then the samples were extracted using a bead shocker in a 2 mL tube with 5 mm zirconia beads and 80% MeOH for 2 min at 1,000 rpm (Shake Master NEO, Biomedical Sciences). The extracted solutions were centrifuged at 10^4^ g for 1 min, and 100 μL centrifuged solution and 10 μL 2 mg/L [UL-^13^C_6_^glc^]-Suc (omicron Biochemicals, USA) were dispensed in a 1.5 mL tube. After drying the solution using a centrifuge evaporator (Speed vac, Thermo), 100 μL Mox regenet (2% methoxyamine in pyridine, Thermo) was added to the 1.5 mL tube, and the metabolites were methoxylated at 30°C and 1,200 rpm for approximately 6 h using a thermo shaker (BSR-MSC100, Biomedical Sciences). After methoxylation, 50 μL 1% v/v of trimetylchlorosilane (TMS, Thermo) was added to the 1.5 mL tube. For TMS derivatization, the mixture was incubated for 30 min at 1,200 rpm at 37°C as mentioned above. Finally, 50 μL the derivatized samples were dispensed in vials for GC-QqQ-MS analyses (AOC-5000 Plus with GCMS-TQ8040, Shimadzu Corporation). Suc and [UL-^13^C_6_^glc^]-Suc were detected in the multiple reaction monitoring (MRM) mode. MRM transitions were Suc-8TMS, 361.0 > 73.0; [UL-^13^C_6_^glc^]-Suc-8TMS, 367.0 >174.0 (parent > daughter). The GC-QqQ-MS analyses were carried out as follows: GC column, BPX-5 0.25 mm I.D. df = 0.25 μm × 30 m (SGE); insert, split insert with wool (RESTEK); temperature of GC vaporization chamber, 250°C; gradient condition of column oven, 60°C for 2 min at the start and 15°C/min to 330 for 3 min of hold; injection mode, split (1:30); carrier gas control, 39 cm^2^/s; interface temperature of MS, 280°C; ion source temperature of MS, 200°C; loop time of data collection, 0.25 second. Raw data collection and calculation of the GC-MS peak area values were carried out using GCMS software solution (Shimadzu Corp., Kyoto, Japan). Suc contents were quantified per etiolated seedling using the internal standard method.

### Quantification of IAA

Endogenous IAA was extracted with 80% (v/v) acetonitrile containing 1% (v/v) acetic acid from whole WT and mutant seedlings after freeze-drying. IAA was purified using a solid-phase extraction column (Oasis WAX, Waters Corporation, Milford, MA, USA) and the IAA levels were determined using a quadrupole/time-of-flight tandem mass spectrometer (Triple TOF 5600, SCIEX, Concord, Canada) coupled with the Nexera UPLC system (Shimadzu Corp., Kyoto, Japan). Conditions for purification, LC and MS/MS analyses were previously described [74].

### Statistical analyses

In this study, the statistical analyses included the Student’s *t*-test, Dennett’s test, or Tukey’s honestly significant difference (HSD) test (R ver. 3.5.1; [75]). Multiple comparisons were performed using the multcomp package [76]. A bee swarm plot was created using the beeswarm package [77].

## Supporting information

S1 Fig Effect of Altered IBA metabolism or Transport on Compensated Cell Enlargement in *fugu5–1*. (A) Seedling gross phenotypes (left panels) and corresponding images of palisade tissue cells (right panels) of plants grown on rockwool. Seedling photographs were taken at 10 DAS. Bar = 2 mm. Palisade tissue cell images were taken at 25 DAS. Bar = 50 μm. (B) Data show cell numbers, cell areas, and cotyledon areas of the indicated genotypes. Cotyledons of each mutant were dissected from plants grown on rockwool for 25 DAS, fixed in FAA, and cleared for microscopic observations. Data are means ± SD (n = 8 cotyledons). Single asterisk indicates that the mutant was statistically significantly different compared to the WT (Student’s t-test at *P* < 0.001, Bonferroni correction). Double asterisk indicates that the double mutant has a statistically significant difference compared to *fugu5–1* (Student’s t-test at *P* < 0.001, Bonferroni correction). DAS, days after sowing.
(TIFF)

S2 Fig Exogenous Supply of Sucrose Cancelled Compensation. Data represent cotyledon cell numbers, cotyledon cell areas and cotyledon areas. Cotyledons of each genotype were dissected from plants grown on MS medium for 25 DAS with 2% Suc, fixed in FAA, and cleared for microscopic observations. Data are means ± SD (n = 8 cotyledons). Single asterisk indicates that the mutant was statistically significantly different compared to the WT (Dunnett’s test at *P* < 0.01; R version 3.5.1). DAS, days after sowing.
(TIFF)

S3 Fig Gross Phenotype of Vegetative and Reproductive Stages. Plant gross phenotypes at the vegetative stage (left panels) and reproductive stage (right panels) of the indicated genotypes. Photographs were taken at 21 DAS (left panels). Bar = 1 cm; or at 37 DAS (right panels). Bar = 5 cm. DAS, days after sowing.
(TIFF)

S4 Fig Effect of *ibr* Mutations on Cotyledon Cellular Phenotypes. **(A)** Plant gross phenotype taken at 10 DAS. Bar = 2 mm (upper panels). Bar = 5 mm (lower panels). **(B)** Cotyledon aspect-ratio in WT and ibr mutants. Data in the beeswarm plots indicate the value of aspect-ratio (n ≦ 9 cotyledons). **(C)** Data represent cotyledon cell numbers, cell areas and cotyledon area of mutants with defects in IBA-to-IAA conversion. Data are means ±SD (n = 8 cotyledons). Single asterisk indicates that the mutant was statistically significantly different compared to the WT (Dunnett’s test at *P* < 0.01; R version 3.5.1). DAS, days after sowing.
(TIFF)

S5 Fig Compensated Cell Enlargement in the *ibr10–2* Mutant is Suppressed by *ech2–1*. **(A)** Cell numbers, cell areas, and cotyledons areas, respectively, of plants grown on rockwool for 25 DAS. Data are means ± SD (n = 8 cotyledons). Single asterisk indicates that mutants were statistically significantly different compared to the WT (Dunnet’s test at *P* < 0.05; R version 3.5.1). (**B)** Cell numbers, cell areas, and cotyledons areas, respectively, of plants grown on MS medium supplied with 2% Suc for 25 DAS. Data are means ± SD (n = 8 cotyledons). Single asterisk indicates that mutants were statistically significantly different compared to the WT (Dunnet’s test at *P* < 0.01; R version 3.5.1). DAS, days after sowing.
(TIFF)

S6 Fig Suc Contents in Etiolated Seedlings of *ibr1–2* and *ibr3–1*. Suc content quantification using the internal standard methods of GC-QqQ-MS for 100 etiolated seedlings after growth on MS medium without Suc for three days after induction of seed germination (DAI). Data are means ± SD (n = six independent experiments; three independent measurements per experiment). Single asterisk indicates that the mutant was statistically significantly different compared to the WT (*P* < 0.05 by Dunnett’s test; R version 3.5.1).
(TIFF)

S7 Fig Cotyledon Cellular Phenotypes in Quintuple Mutants. **(A)** Microscopic images of palisade tissue taken at 25 DAS. Bars = 100 μm. **(B)** Cotyledon cell numbers, cotyledon cell areas and cotyledon areas. Cotyledons from each genotype were dissected from plants grown on rockwool for 25 DAS, fixed in FAA, and cleared for microscopic observations. Data are means ± SD (n = 8 cotyledons). Single asterisk indicates that the mutant was statistically significantly different compared to the WT (Dunnett’s test at *P* < 0.05; R version 3.5.1). DAS, days after sowing.
(TIFF)

S8 Fig Endogenous Concentration of IAA in *fugu5–1* and mutant lines with suppressed CCE. Quantification of endogenous IAA in mutant lines with suppressed CCE. Cotyledons of the indicated lines were collected at 10 DAS. Data are means ± SD (n = 3 independent experiments). NS, not significant (*P* < 0.05 by Dunnett’s test; R version 3.5.1). DAS, days after sowing.
(TIFF)

S9 Fig Effect of Failure of IBA-to-IAA Conversion and IBA Efflux on Compensated Cell Enlargement. **(A)** Cotyledon cell numbers, cotyledon cell areas and cotyledon areas in double mutants with defects in IBA-to-IAA conversion and extracellular export of IBA. Data are means ± SD (n = 8 cotyledons). Single asterisk indicates that the mutant was statistically significantly different compared to the WT (Student’s t- test at *P* < 0.05, Bonferroni corrected). **(B)** Cotyledon cell numbers, cotyledon cell areas and cotyledon areas in the *fugu5–1* background double mutants involved in IBA-to-IAA conversion and extracellular export of IBA. Data are means ± SD (n = 8 cotyledons). Single asterisk indicates that the mutant was significantly different compared to the WT (Student’s t- test at *P* < 0.05, Bonferroni corrected).
(TIFF)

S1 Table List of Oligonucleotide Primers used in this Study. (TIFF)

S1 Data Numerical Data. (XLSX)
