# Supplementary figures and images for: An auxin signaling network translates low-sugar-state input into compensated cell enlargement in the fugu5 cotyledon
Source: PLoS Genet. 2021 Aug 5;17(8):e1009674. doi: 10.1371/journal.pgen.1009674 (PMC8341479; doi:10.1371/journal.pgen.1009674)

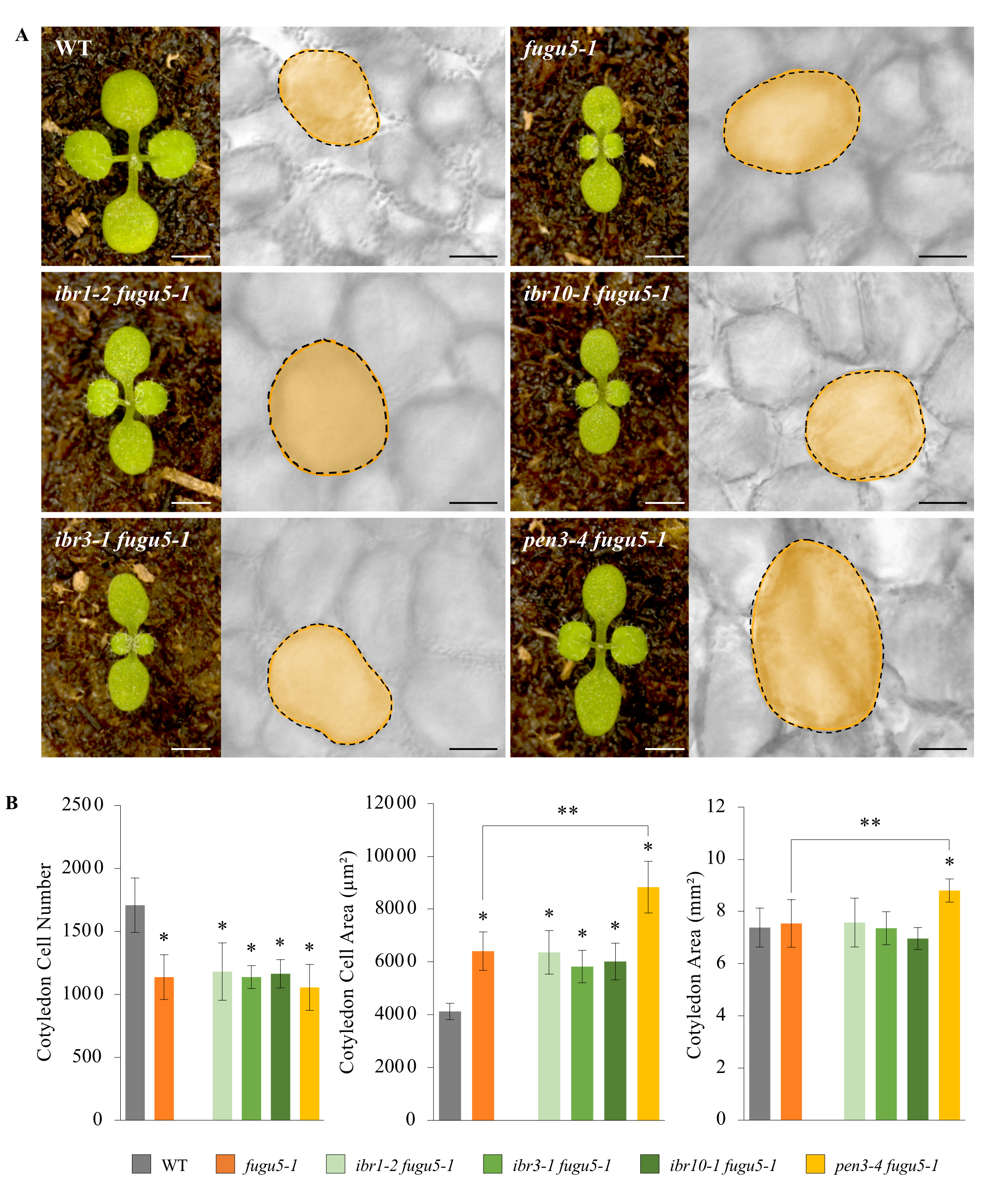

Supplement: S1 Fig — (A) Seedling gross phenotypes (left panels) and corresponding images of palisade tissue cells (right panels) of plants grown on rockwool. Seedling photographs were taken at 10 DAS. Bar = 2 mm. Palisade tissue cell images were taken at 25 DAS. Bar = 50 μm. (B) Data show cell numbers, cell areas, and cotyledon areas of the indicated genotypes. Cotyledons of each mutant were dissected from plants grown on rockwool for 25 DAS, fixed in FAA, and cleared for microscopic observations. Data are means ± SD (n = 8 cotyledons). Single asterisk indicates that the mutant was statistically significantly different compared to the WT (Student’s t-test at P < 0.001, Bonferroni correction). Double asterisk indicates that the double mutant has a statistically significant difference compared to fugu5–1 (Student’s t-test at P < 0.001, Bonferroni correction). DAS, days after sowing. (TIFF) [file pgen.1009674.s001.tiff]

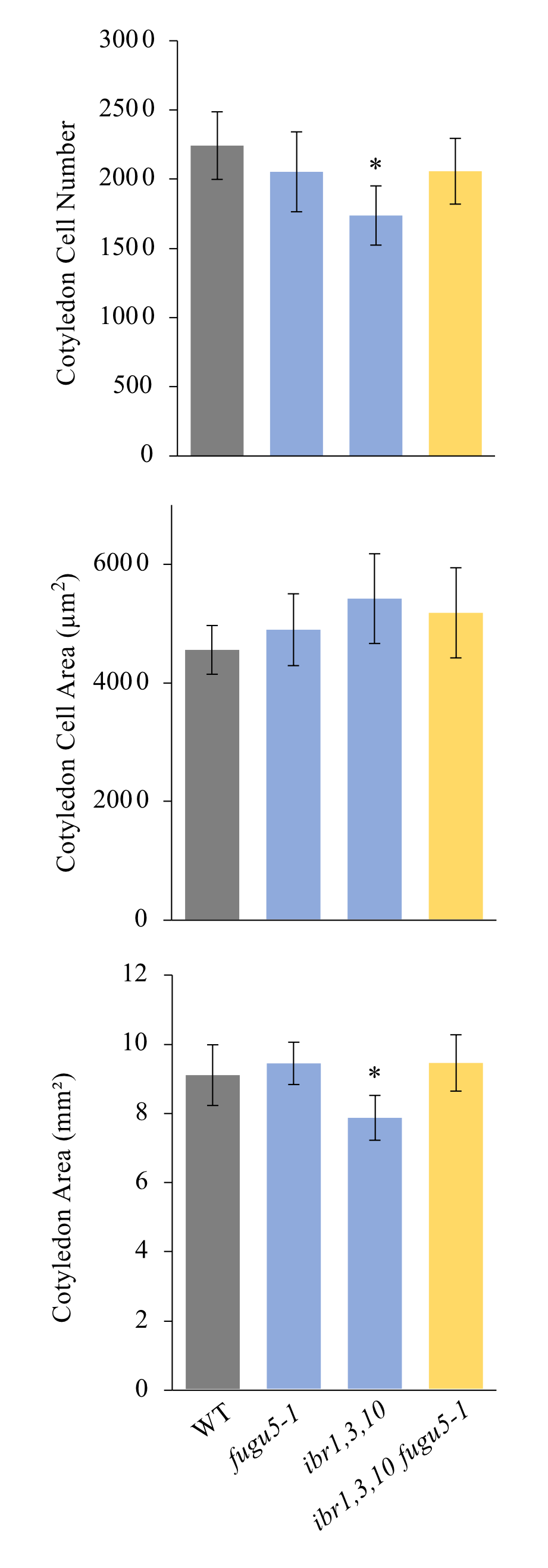

Supplement: S2 Fig — Data represent cotyledon cell numbers, cotyledon cell areas and cotyledon areas. Cotyledons of each genotype were dissected from plants grown on MS medium for 25 DAS with 2% Suc, fixed in FAA, and cleared for microscopic observations. Data are means ± SD (n = 8 cotyledons). Single asterisk indicates that the mutant was statistically significantly different compared to the WT (Dunnett’s test at P < 0.01; R version 3.5.1). DAS, days after sowing. (TIFF) [file pgen.1009674.s002.tiff]

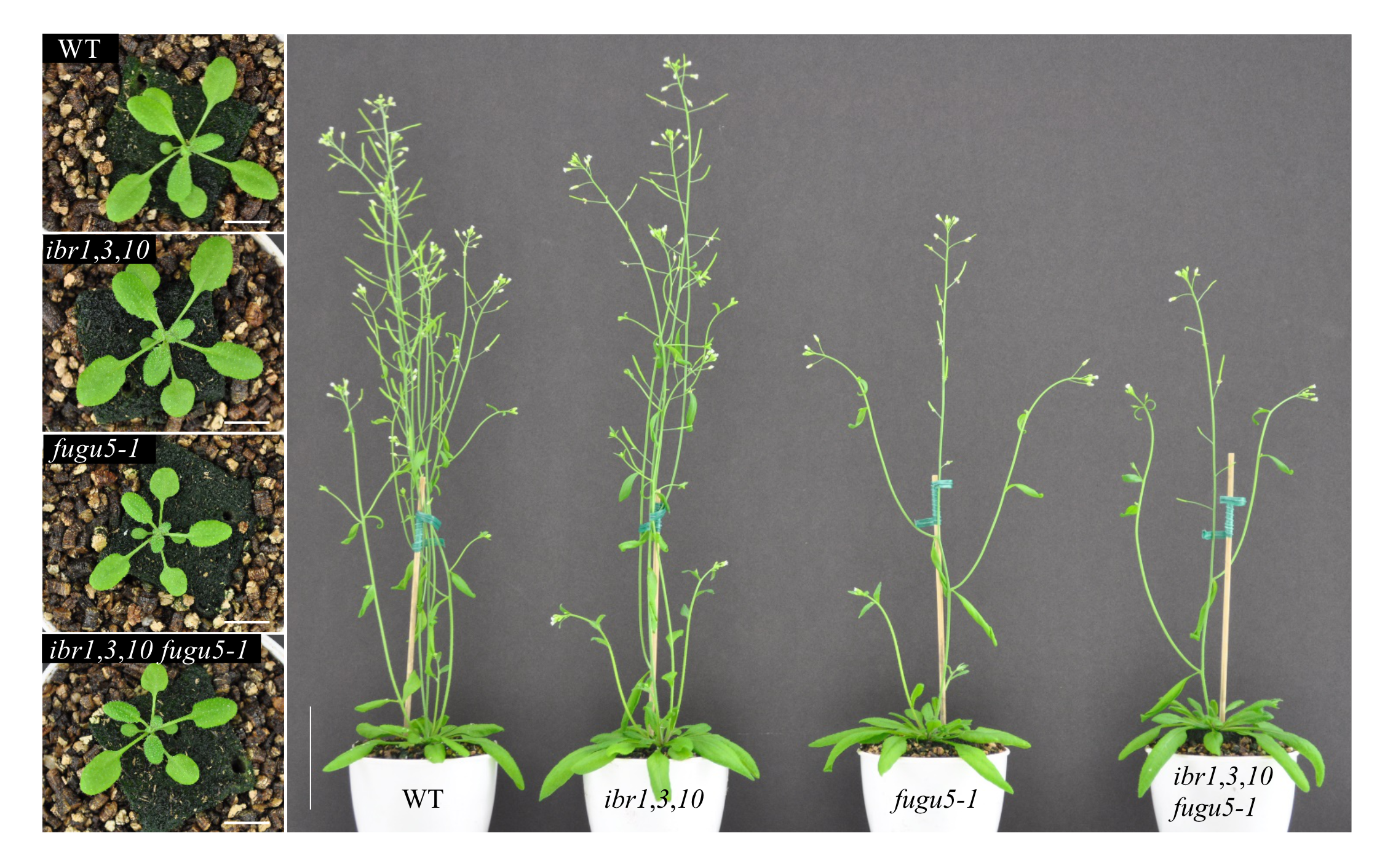

Supplement: S3 Fig — Plant gross phenotypes at the vegetative stage (left panels) and reproductive stage (right panels) of the indicated genotypes. Photographs were taken at 21 DAS (left panels). Bar = 1 cm; or at 37 DAS (right panels). Bar = 5 cm. DAS, days after sowing. (TIFF) [file pgen.1009674.s003.tiff]

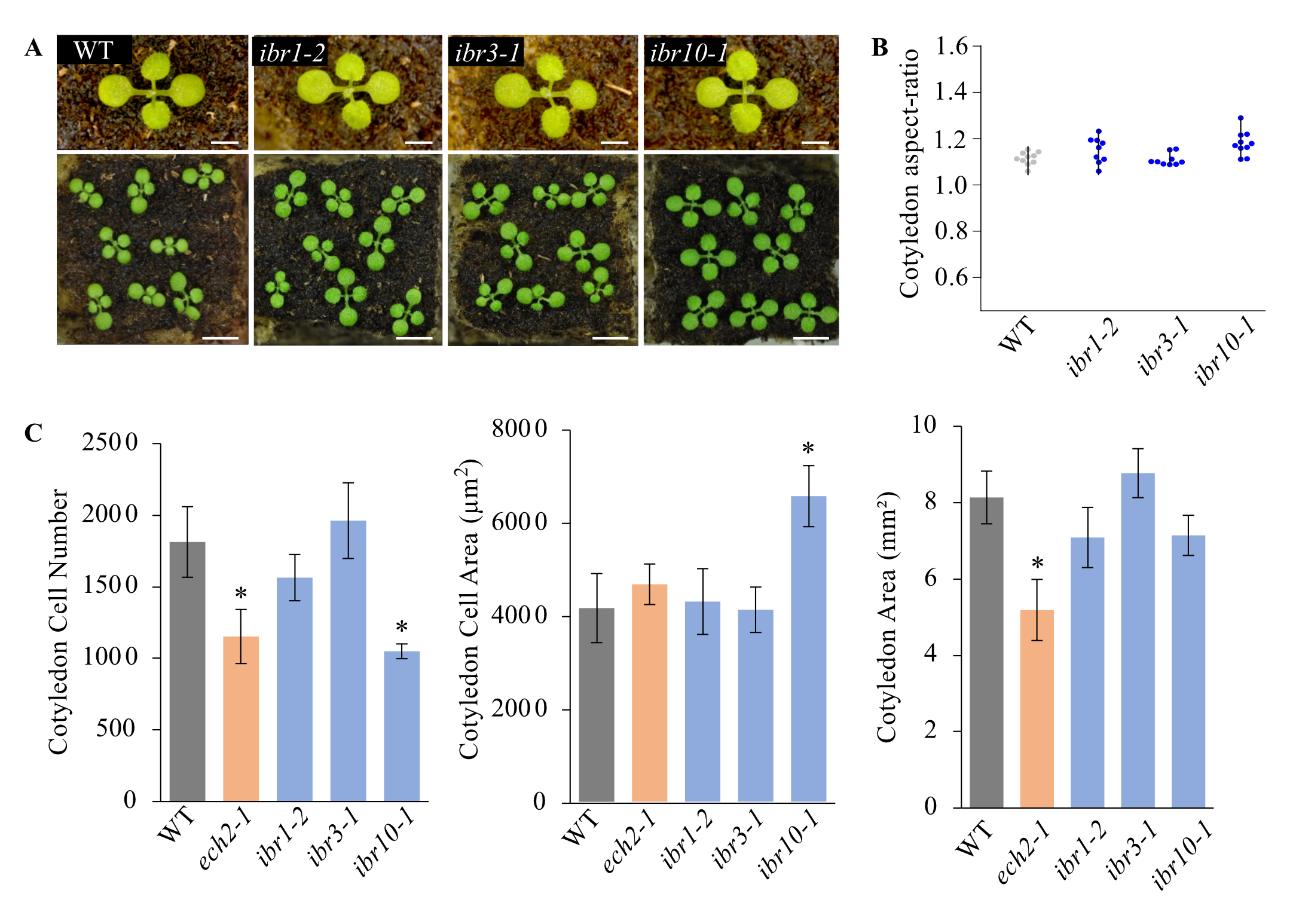

Supplement: S4 Fig — (A) Plant gross phenotype taken at 10 DAS. Bar = 2 mm (upper panels). Bar = 5 mm (lower panels). (B) Cotyledon aspect-ratio in WT and ibr mutants. Data in the beeswarm plots indicate the value of aspect-ratio (n ≦ 9 cotyledons). (C) Data represent cotyledon cell numbers, cell areas and cotyledon area of mutants with defects in IBA-to-IAA conversion. Data are means ±SD (n = 8 cotyledons). Single asterisk indicates that the mutant was statistically significantly different compared to the WT (Dunnett’s test at P < 0.01; R version 3.5.1). DAS, days after sowing. (TIFF) [file pgen.1009674.s004.tiff]

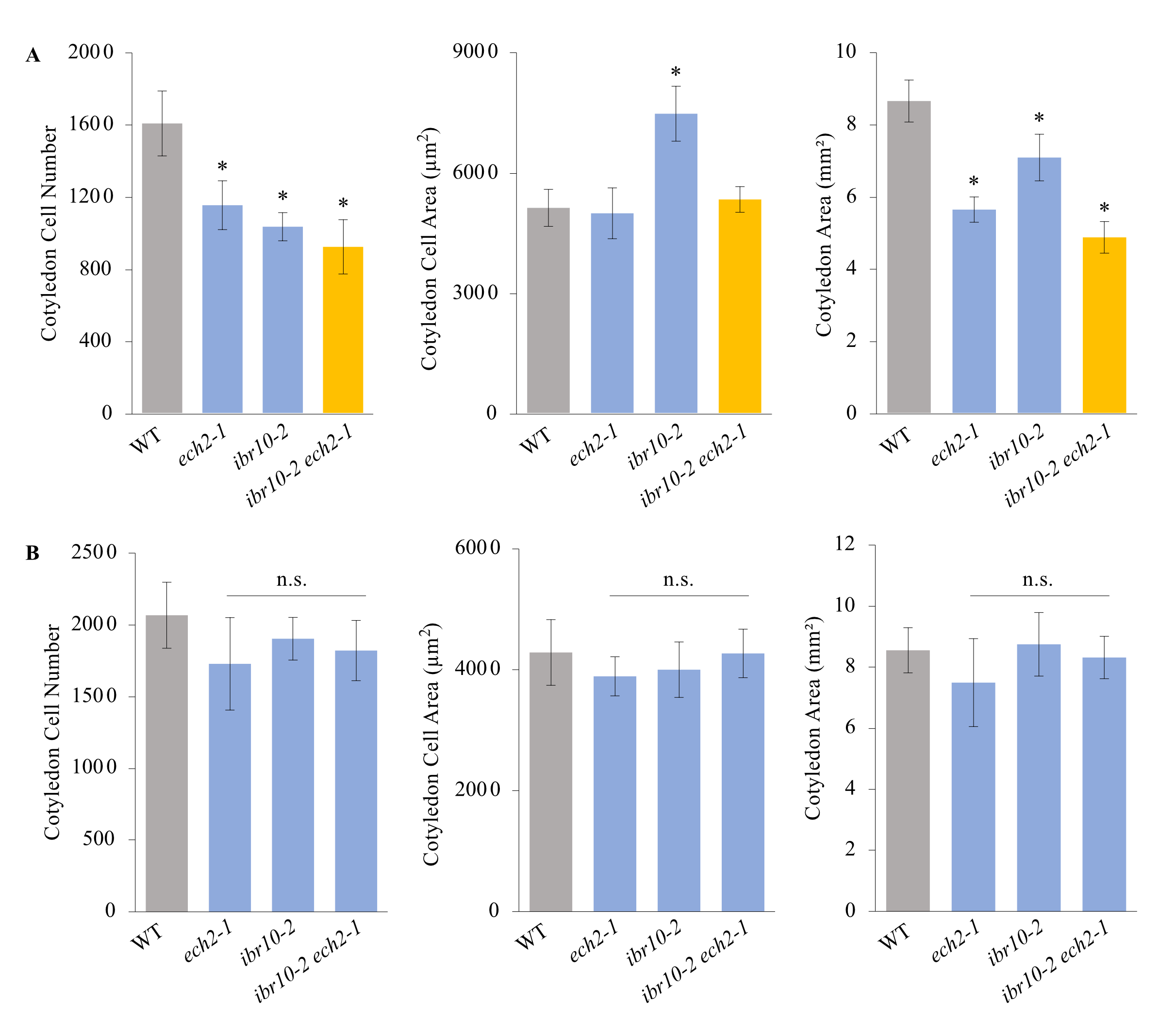

Supplement: S5 Fig — (A) Cell numbers, cell areas, and cotyledons areas, respectively, of plants grown on rockwool for 25 DAS. Data are means ± SD (n = 8 cotyledons). Single asterisk indicates that mutants were statistically significantly different compared to the WT (Dunnet’s test at P < 0.05; R version 3.5.1). (B) Cell numbers, cell areas, and cotyledons areas, respectively, of plants grown on MS medium supplied with 2% Suc for 25 DAS. Data are means ± SD (n = 8 cotyledons). Single asterisk indicates that mutants were statistically significantly different compared to the WT (Dunnet’s test at P < 0.01; R version 3.5.1). DAS, days after sowing. (TIFF) [file pgen.1009674.s005.tiff]

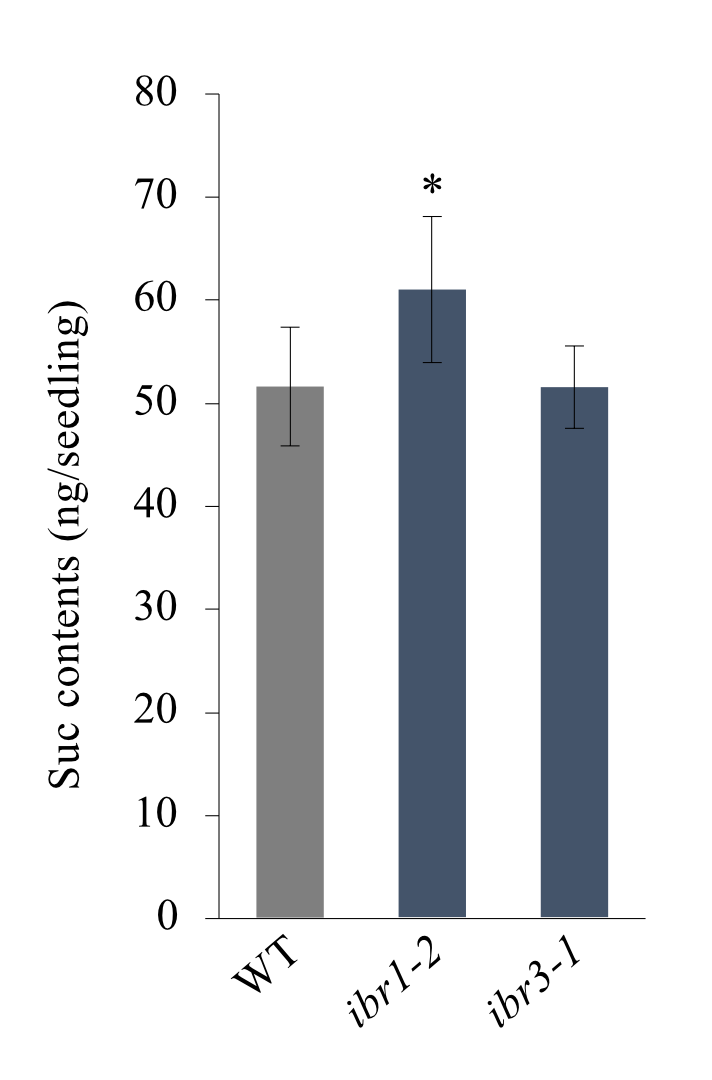

Supplement: S6 Fig — Suc content quantification using the internal standard methods of GC-QqQ-MS for 100 etiolated seedlings after growth on MS medium without Suc for three days after induction of seed germination (DAI). Data are means ± SD (n = six independent experiments; three independent measurements per experiment). Single asterisk indicates that the mutant was statistically significantly different compared to the WT (P < 0.05 by Dunnett’s test; R version 3.5.1). (TIFF) [file pgen.1009674.s006.tiff]

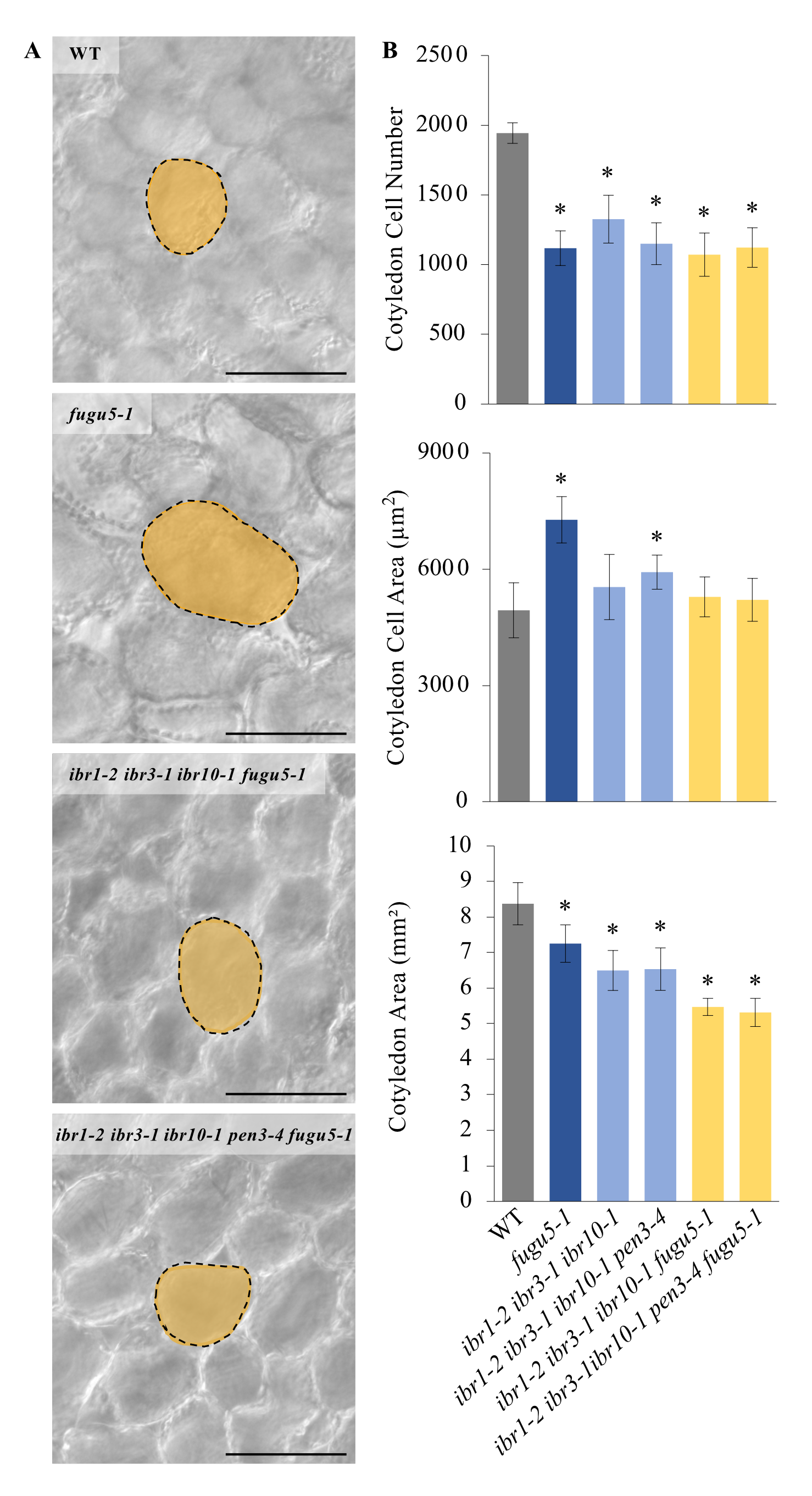

Supplement: S7 Fig — (A) Microscopic images of palisade tissue taken at 25 DAS. Bars = 100 μm. (B) Cotyledon cell numbers, cotyledon cell areas and cotyledon areas. Cotyledons from each genotype were dissected from plants grown on rockwool for 25 DAS, fixed in FAA, and cleared for microscopic observations. Data are means ± SD (n = 8 cotyledons). Single asterisk indicates that the mutant was statistically significantly different compared to the WT (Dunnett’s test at P < 0.05; R version 3.5.1). DAS, days after sowing. (TIFF) [file pgen.1009674.s007.tiff]

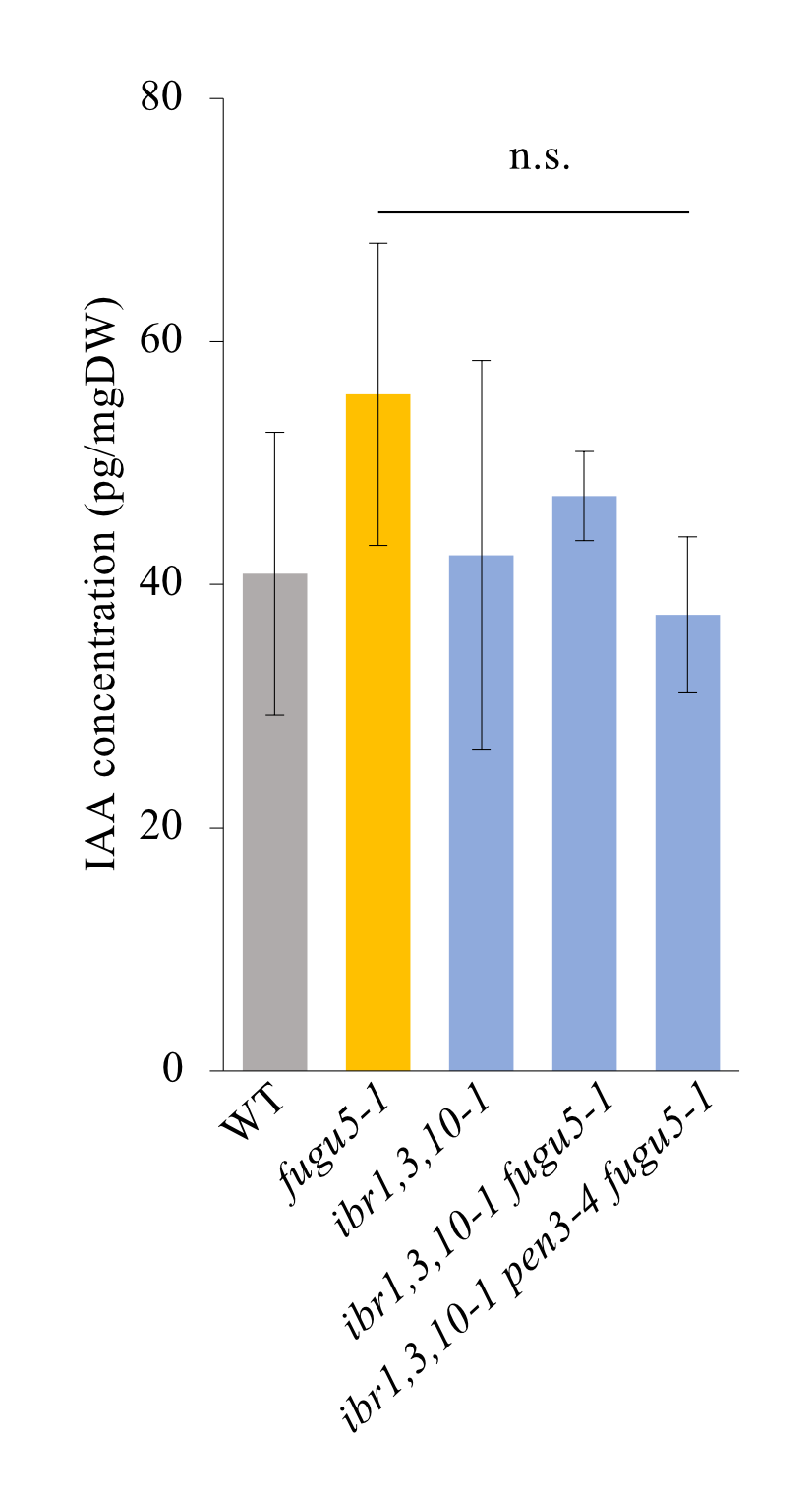

Supplement: S8 Fig — Quantification of endogenous IAA in mutant lines with suppressed CCE. Cotyledons of the indicated lines were collected at 10 DAS. Data are means ± SD (n = 3 independent experiments). NS, not significant (P < 0.05 by Dunnett’s test; R version 3.5.1). DAS, days after sowing. (TIFF) [file pgen.1009674.s008.tiff]

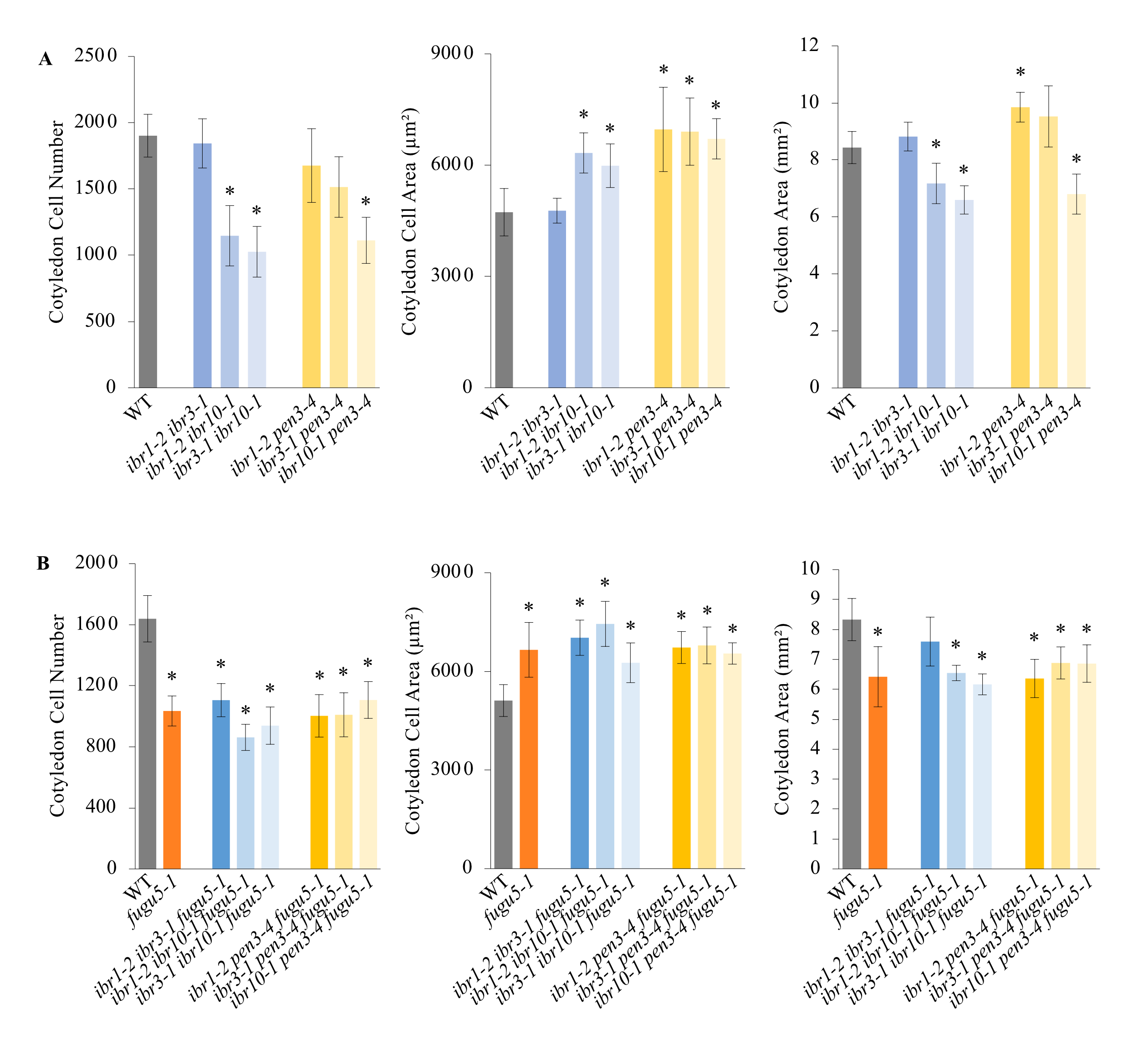

Supplement: S9 Fig — (A) Cotyledon cell numbers, cotyledon cell areas and cotyledon areas in double mutants with defects in IBA-to-IAA conversion and extracellular export of IBA. Data are means ± SD (n = 8 cotyledons). Single asterisk indicates that the mutant was statistically significantly different compared to the WT (Student’s t- test at P < 0.05, Bonferroni corrected). (B) Cotyledon cell numbers, cotyledon cell areas and cotyledon areas in the fugu5–1 background double mutants involved in IBA-to-IAA conversion and extracellular export of IBA. Data are means ± SD (n = 8 cotyledons). Single asterisk indicates that the mutant was significantly different compared to the WT (Student’s t- test at P < 0.05, Bonferroni corrected). (TIFF) [file pgen.1009674.s009.tiff]
